# Synthesis and molecular docking of novel pyrazole, pyrimidine, and pyridine derivatives as potent antimicrobial, antibiofilm, and anticancer agents

**DOI:** 10.1038/s41598-026-65144-w

**Published:** 2026-08-08

**Authors:** A. M. A. Hassan, E. S. Essam, Selwan Hamed, M. H. Helal, A. K. El-Ziaty, Rania S. Ali

**Affiliations:** 1 Department of Chemistry, Faculty of Science, Ain Shams University, Abbassia, Cairo, 11566 Egypt; 2 Department of Basic Science, Faculty of Technology and Education, Capital University, Cairo, Egypt; 3 Department of Microbiology and Immunology, Faculty of Pharmacy, Capital University, Ain Helwan, Helwan, 11795 Egypt; 4 Department of Chemistry, Faculty of Science, Capital University, Ain Helwan, Cairo, Egypt

**Keywords:** Chalcone, Pyrazole, Oxazinone, Pyrimidine, Antimicrobial, Antibiofilm, Biochemistry, Biotechnology, Cancer, Chemical biology, Chemistry, Drug discovery, Microbiology

## Abstract

The development of novel antimicrobial and anticancer agents remains a priority due to rising drug resistance and high systemic toxicity of current treatments. A series of novel pyrazole (**2–6**), pyrimidine (**7–10**), and pyridine/oxazinone (**11–14**) derivatives were synthesized from a chalcone scaffold (**1**). They were evaluated for antimicrobial, antibiofilm (*Pseudomonas aeruginosa*), and cytotoxic (HepG2 cells) activities. Molecular docking and qRT-PCR were performed to study their mechanism. Pyrazoles **3–5** and oxazinone **13** showed potent antibacterial activity against *S. aureus* (MIC = 2–3.12 µg/mL). Compounds **4** and **13** effectively eradicated *P. aeruginosa* biofilms, achieving a ≥ 5 log_10_ reduction within 4 h at 0.8 × MIC, driven by disruption of the bacterial respiratory chain. For anticancer activity, compounds **10** and **13** selectively reduced HepG2 cell viability to 35–40% *via* oxidative stress-mediated apoptosis. Mechanistically, compound **4** reduced gyrB expression in *E. coli* by approximately 4.6-fold, while molecular docking supported its interaction with the ATP-binding pocket of DNA gyrase. Overall, the functionalized heterocycles, particularly compounds **4** and **13**, represent promising dual-acting candidates with potent antibacterial, antibiofilm, and selective anticancer activities.

**Supplementary Information:**

The online version contains supplementary material available at 10.1038/s41598-026-65144-w.

## Introduction

**Fig. 1:**
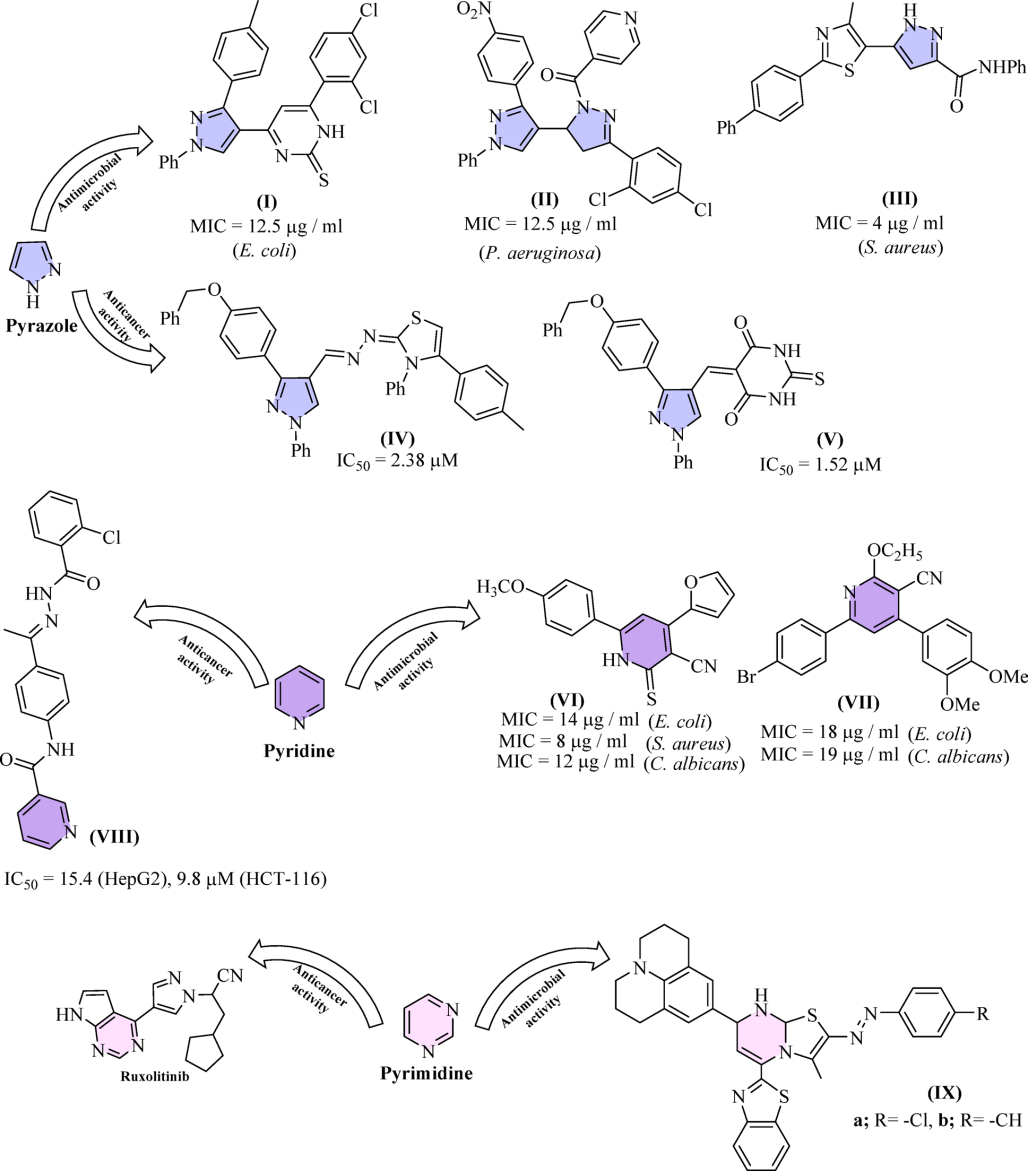
Antimicrobial and anticancer compounds containing the pyrazole, pyridine, and pyrimidine scaffold.

Nitrogen-containing rings are essential for simulating real biomolecules and controlling important pharmacological pathways in heterocyclic scaffolds, which are the foundation of contemporary medicinal chemistry. The resistance of microbial strains to classic drugs synthesized in recent years is one of the central medical problems. Therefore, one of the research directions of chemists is the synthesis of new compounds with antimicrobial properties. The pyrazole derivatives showed antifungal activity against *Aspergillus niger*, *Candida albicans*, and *Rhodotorula glutinis*, and antimicrobial activity against *Escherichia coli*, *Proteus mirabilis*, and *Staphylococcus albus* strains^1,2^. For substance, pyrazole derivative **I** found is a potent inhibitor of *E. coli* with an MIC value of 12.5 μg/ml. While compound **II** was found as a moderate growth inhibitors of *S. aureus* bacteria, compound **III** was a potent anti-MRSA agents^3^ with MIC values as low as 4 μg/ml, as shown in Fig. 1. Additionally, anticancer, antiviral, antidiabetic, anti-hypertensive, and antimicrobial actions are among the many properties of the pyrazolic nucleus^4–23^. Synthesized pyrazoles^24^ were investigated for their ability to inhibit various HepG2, UO-31, and HCT116 cancer cell lines. For example, compounds **IV** and **V** showed the strongest anticancer effects among the substances that were studied. In contrast to compound **V**, which exhibited IC_50_ values of 1.2 µM, 1.8 µM, and 1.5 µM, respectively, compound **IV** demonstrated IC_50_ values of 1.5 µM against HCT116, 2.0 µM against UO-31, and 1.8 µM against HepG2.

Additionally, pyridine and its derivatives are useful heterocyclic chemicals that are essential to many medicinal applications^25^. Pyridine and its derivatives improve solubility and bioavailability parameters since they are polar and ionizable aromatic compounds^26^. Numerous pyridine derivatives have been studied for their antibacterial properties. Compound **VI** exhibited activity against *E. coli* and *S. aureus* bacteria with an MIC value of 14 μg/ml and 8 μg/ml, respectively, and against *C. albicans* fungi with an MIC value of 12 μg/ml^27^. Whereas compound **VII** showed growth inhibition zones for *E. coli* and *C. albicans* with an MIC value of 18 μg/ml and 19 μg/ml, respectively^28^. Synthesized pyridines as compound **VIII** revealed the highest anti-proliferative activities with IC_50_ values of 15.4 and 9.8 µM against HCT-116 and HepG2, respectively^29^, as shown in Fig. 1.

Furthermore, the therapeutic applications of pyrimidine derivatives are well known in medicinal chemistry^30^. Pyrimidine derivatives showed significant antimicrobial activity against both bacterial and fungal strains. For instance, compounds **IXa, b** emerged as the most potent antimicrobial agents, exhibiting strong antibacterial activity against *S. aureus* with MIC values of 6.5 and 6.8 μM, respectively, as shown in Fig. 1. These compounds also demonstrated significant antifungal activity against *C. albicans* with MIC values in the range of 26.2–27.1 μM^31^. According to reports, condensed pyrimidine derivatives have antihypertensive^32^, and central nervous system effects^33^. Additionally, pyrimidines have been shown to be dual EGFR/VGFR2 inhibitors in the past^34,35^, antibacterial^36^, and antibiofilm^37^. According to the previously mentioned literature, many pyrazole, pyridine, and pyrimidine derivatives still have drawbacks like moderate potency, a lack of structural diversity, or inadequate dual-acting capabilities (e.g., simultaneous antibacterial, antibiofilm, and anticancer actions). We devised and synthesized a unique series of functionalized heterocycles integrated with both a lipophilic 2-furyl moiety and an electron-withdrawing *p*-nitrophenyl ring on a chalcone core in order to overcome these difficulties and improve therapeutic efficacy. The structural architecture of our newly designed pyrazoles, pyrimidines, and fused pyridioxazinones was strategically engineered to promote superior cell-wall penetration, target bacterial DNA gyrase more effectively, and selectively induce oxidative stress-mediated apoptosis in cancer cells, in contrast to previously reported derivatives (such as compounds **I**-**IXb**, which frequently lack this specific synergistic combination). In this article, we describe their thorough synthesis, molecular docking simulations, and assessment as effective antibacterial, antibiofilm, and specific anticancer medicines.

## Results and discussion

### Chemistry

**Scheme 1:**
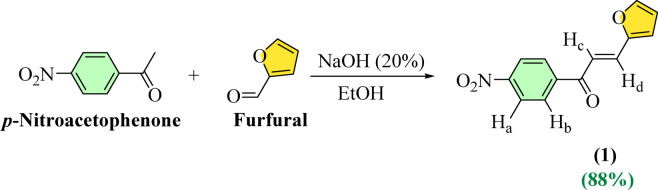
Synthesis of 3-(furan-2-yl)-1-(4-nitrophenyl)prop-2-en-1-one (**1**).

The previously reported *α*,*β*-unsaturated ketone 3-(furan-2-yl)-1-(4-nitrophenyl)prop-2-en-1-one **(1)**^38^ was utilized, bearing nitro and furyl moieties, as a scaffold to create novel heterocycles with expected pharmaceutical efficiency, building on our previous efforts in the synthesis of pharmaceutically active heterocycles^39–44^. The synthesis of compound** 1** began with furfural and *p*-nitroacetophenone interacting in the presence of (10 ml) of an alcoholic sodium hydroxide solution (20%) under a stirring system at room temperature. This produced 3-(furan-2-yl)-1-(4-nitrophenyl)prop-2-en-1-one (**1**) smoothly in a noticeable yield (88%) as yellow crystals. The production of the building block *α*,*β*-unsaturated ketone was depicted in Scheme 1.

The chemical structure of compound **1** was described using varied spectroscopic studies. The FT-IR spectrum showed absorption bands at *v* 1587 and 1659 cm^−1^, which were identified as belonging to the C = C and C = O groups. With two doublet signals at *δ* 7.61 and 7.49 ppm, respectively, associated with methylene protons (H_**d**_ and H_**c**_), the ^1^H-NMR spectrum maintained its predicted structure. Compound **1** underwent interactions with several nitrogen nucleophiles in order to accomplish the study’s intended purpose, which is the creation of antimicrobial active heterocycles.

In an ethanol solvent, hydrazine was added to chalcone **1** by Aza-Michael. This was followed by 1,5-exo-trig cyclization to produce the pyrazole derivative **2**^45^, whose structure was revealed by spectral analysis, such as the FT-IR spectrum, which showed absorption bands at v 3335 and 1593 cm^−1^, corresponding to the NH and C = N groups, respectively. The proton of the NH group was represented by a singlet signal in the ^1^H-NMR spectrum at *δ* 7.18 ppm, which vanished after deuteration. Furthermore, the protons of the (CH) and CH₂ groups of the pyrazole ring are represented by triplet and multiplet signals at *δ* 4.99 and 3.40–3.07 ppm, respectively. Additionally, the molecular ion peak was visible in the mass spectrum at m/z = 257 (30.65%). After the chemical structure of pyrazole derivative **2** was confirmed, its nucleophilicity was investigated by looking at how it interacted with an electrophilic carbonyl molecule.

The nucleophilicity of compound **2** was examined through its interaction with ethyl chloroacetate using a catalytic quantity of triethylamine, which produced the *N*-acylpyrazole derivative **3** by nucleophilically adding to the carbonyl carbon of ethyl chloroacetate. FT-IR spectrum of compound **3** showed an absorption band associated with the C = O group at* v* 1693 cm^−1^. Additionally, the nucleophilic addition process rather than the substitution pathway was validated by the ^1^H-NMR spectrum, which showed a singlet peak at *δ* 3.71 ppm corresponding to the protons of the (CH_2_Cl) group with no signals attributable to the ethyl group. A signal associated with the (C = O) group was seen at *δ* 167.21 ppm in the ^13^C-NMR spectrum.

The same 1,2-binucleophile (hydrazine hydrate) was examined, but this time in the presence formic acid, which afforded a novel pyrazole derivative **4**, that bearing formyl group in its structure. The elucidation of the structure of derivative **4** through its FT-IR spectrum that showed an absorption band at *ν* 1672 cm^−1^ attributed to the C = O group. Additionally, ^1^H-NMR spectrum of compound **4** displayed a singlet signal at *δ* 8.89 ppm, corresponding to the proton of the CHO. Also showed a doublet of doublet signal at *δ* 5.68, and 5.72 ppm for CH group in pyrazole ring, and a multiplet signal at *δ* 3.90–3.41 ppm for CH_2_ group. Furthermore, ^13^C-NMR spectrum displayed a signal at *δ* 160.68 ppm related to C = O group. The mass spectrum showed the molecular ion M^**∙ +**^ peak at *m/z* = 285 (22.00%).

**Scheme 2:**
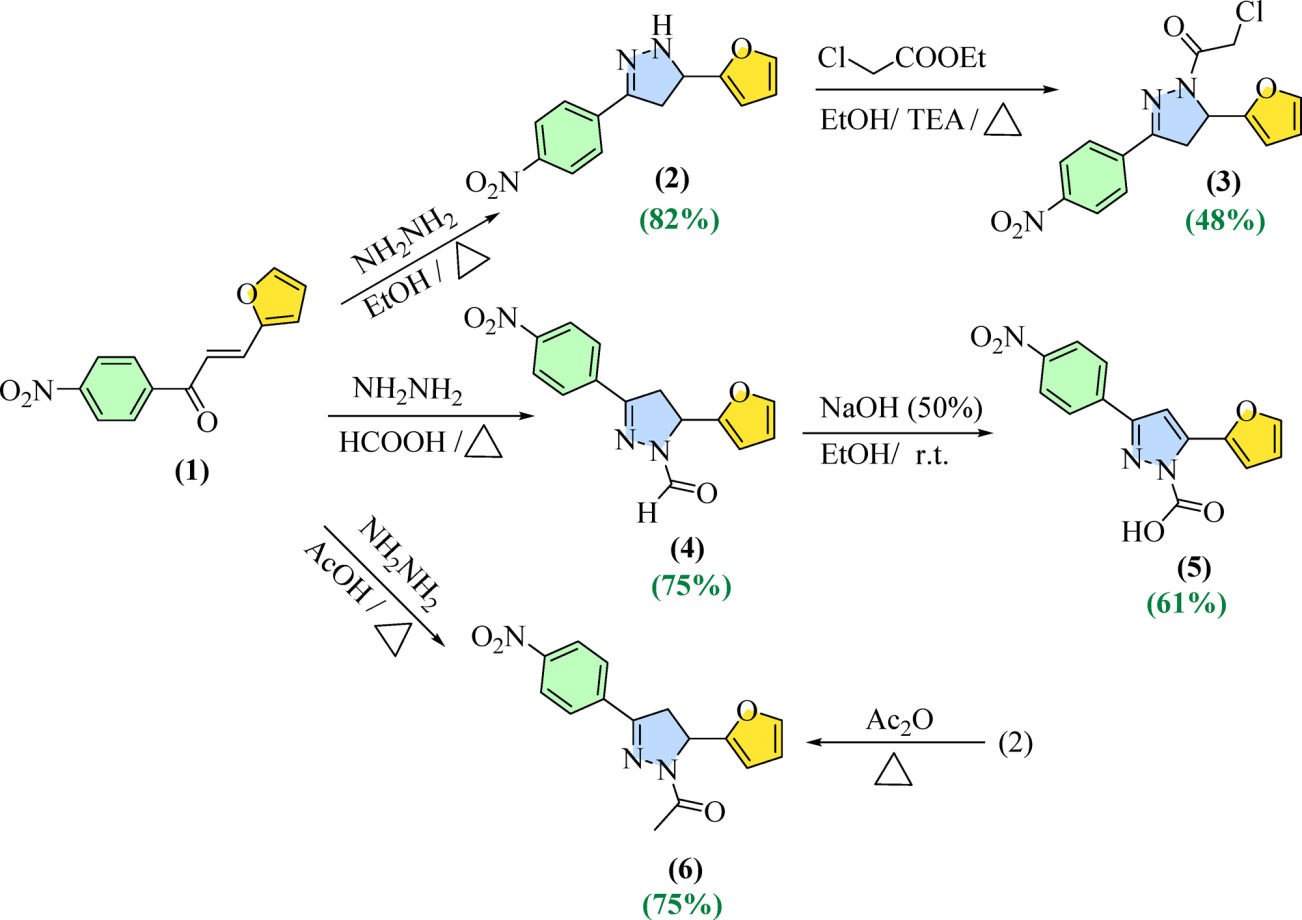
Strategic synthetic routes for the construction of pyrazole derivatives (**2–6**).

The action of an aqueous solution of sodium hydroxide (50%, w/v) on compound **4** afforded a novel pyrazole derivative **5** with full aromatization bearing a carboxylic acid group, as shown in Scheme 2. Its FT-IR spectrum showed absorption bands at *ν* 3441 and 1680 cm^−1^ attributed to OH and C = O groups, respectively. The ^1^H-NMR spectrum displayed a singlet signal at *δ* 13.95 ppm related to COOH group which disappeared upon deuteration, and a singlet at *δ* 7.79 ppm for CH group of pyrazole ring. The ^13^C-NMR spectrum displayed a signal at *δ* 158.72 ppm related to (C = O) group.

Meanwhile, the *N*-acetyl pyrazole derivative **6** was produced from the reaction of hydrazine hydrate with chalcone **1** in the presence of acetic acid^46^. Additionally, this derivative **6** is produced with a notable yield of 75% when compound **4** interacts with acetic anhydride. The structure of pyrazole derivative **6** was confirmed from the FT-IR spectrum analysis which showed the absorption band of carbonyl group at 1658 cm^−1^. The ^1^H-NMR spectrum showed a singlet signal at *δ* 2.29 ppm, corresponding to the proton of the CH_3_ group. Also showed a doublet of doublet signal at *δ* 5.72, and 5.68 ppm for CH group in pyrazole ring, and a multiplet signal at *δ* 3.85–3.39 ppm for CH_2_ group. The mass spectrum showed the molecular ion M^**∙ +**^ peak at *m/z* = 299 (34.12%).

**Scheme 3:**
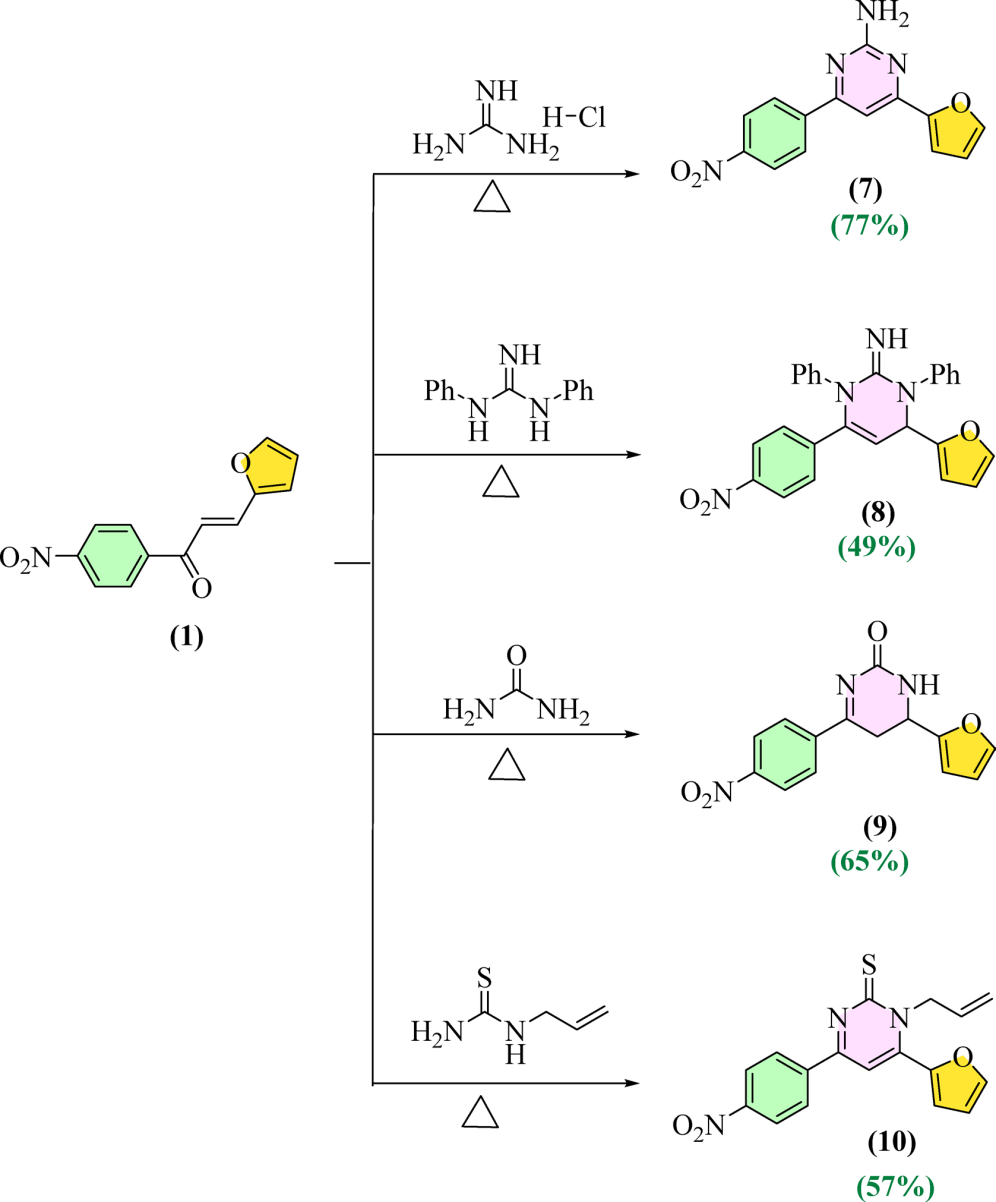
Synthetic pathways of pyrimidine derivatives (**7–10**).

The reactivity of compound **1** also investigated to construct pyrimidine derivatives **(7–10)** via its reaction with various 1,3-binucleophiles such as guanidine hydrochloride, 1,3-diphenylguanidine, urea and *N*-allylthiourea, respectively, in the presence of an alcoholic potassium hydroxide as shown in Scheme 3.

The chemical structures of pyrimidines **7–10** were confirmed from their spectral analysis, the FT-IR spectrum showed the vibrational coupling of NH_2_ group at 3217, 3347 cm^−1^ for pyrimidine derivative **7**^47^ and the NH group of derivative **8** observed at 3200 cm^−1^. The carbonyl group of **9** appeared at 1687 cm^−1^, whereas the thione group of the compound **10** appeared at 1215 cm^−1^. Moreover, the ^1^H-NMR spectrum displayed a singlet signal appeared at *δ* 8.67 ppm for NH group which disappeared upon deuteration for compound **8**. Meanwhile, a singlet signal at *δ* 5.41 ppm for NH group which disappeared upon deuteration for compound **9**. The protons of vinyl group (-CH = CH_2_-) in derivative **10** appeared as a multiplet signal at *δ* 5.05–4.02 ppm. The mass spectra for these derivatives **7–10** showed the molecular ion M^**∙ +**^ peaks at *m/z* = 282 (16.14%), 436 (17.21%), 285 (15.85%), and 339 (13.72%), respectively.

**Scheme 4:**
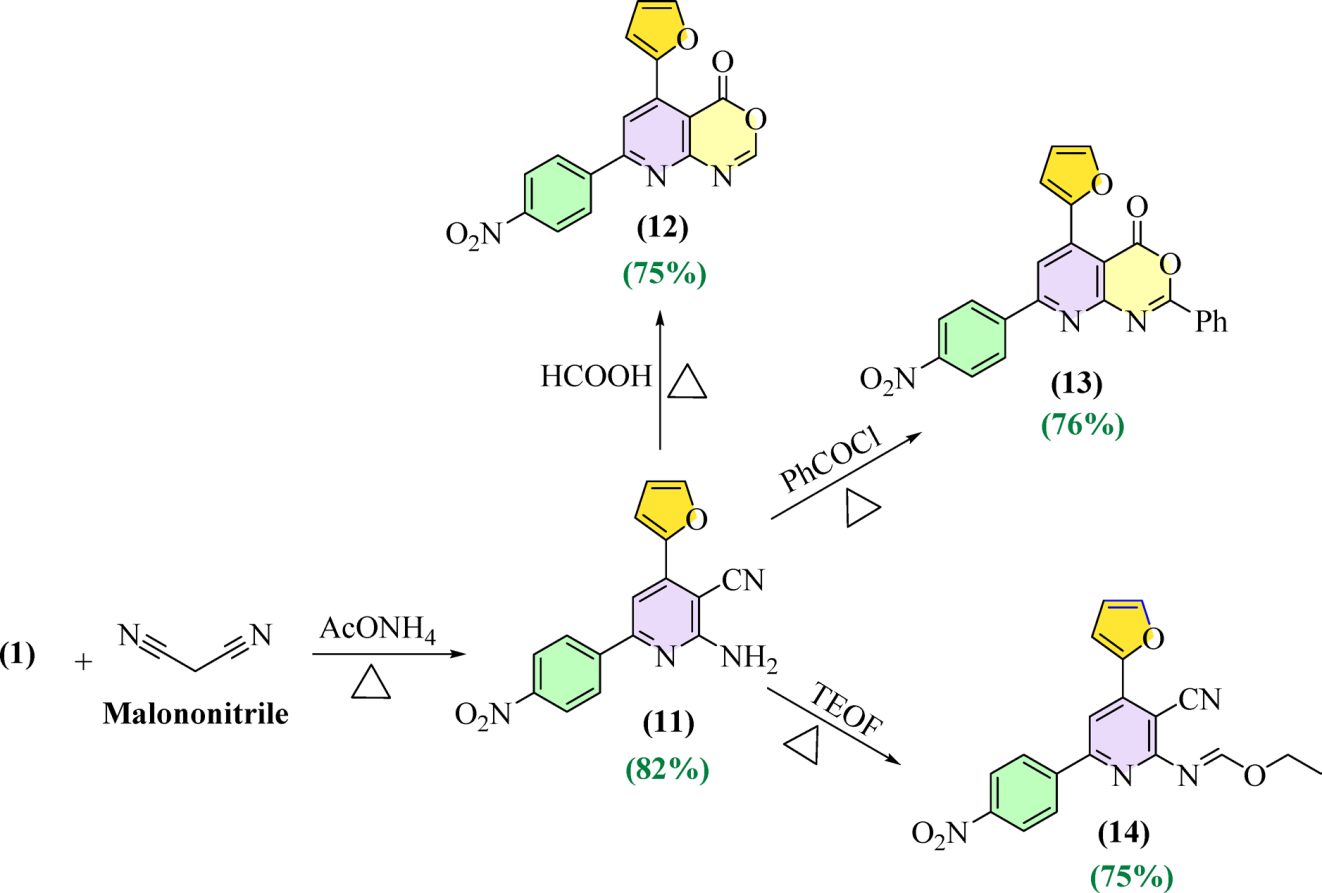
Synthetic pathways of pyridine derivatives (**11–14**).

Our study extended to investigate the interaction of *α*,*β*-unsaturated ketone derivative **1** with carbon nucleophiles. Therefore, the reaction of compound **1** with malononitrile in the presence of ammonium acetate was carried out. This reaction afforded a novel pyridine derivative **11** as an enaminonitrile derivative which bears two significant function groups as C≡N and NH_2_ groups in its structure, which can be used as a precursor for preparation novel pyridines as shown in Scheme 4.

To construct antimicrobial pyridine derivative bearing nitro and furyl moieties, the enaminonitrile **11** was allowed to react with electrophilic reagents such as formic acid, benzoyl chloride and triethyl orthoformate to afford pyridioxazinones **12**, **13** and the pyridine derivative **14**, respectively. The FT-IR spectrum for compound **11** showed absorption bands at *ν* 3353–3241, and 2214 cm^−1^ attributed to NH_2_, and C≡N groups, respectively. Moreover, the ^1^H-NMR spectrum displayed singlet signals at *δ* 7.18 and 6.90 ppm related to the protons of the CH group in pyridine ring, and the NH_2_ group, respectively. The mass spectrum showed the molecular ion M^**∙ +**^ peak at *m/z* = 306 (18.12%).

The interaction of formic acid with the enaminonitrile derivative **11** cyclized to produce the pyrido[2,3-*d*][1,3]oxazinone derivative **12**. This cyclization was supported through the FT-IR spectrum that revealed an absorption peak at *ν* 1714 cm^−1^ related to the formation of the (C = O) group and the absorption peaks for NH_2_ and C≡N groups were not appeared. Again, to confirm this elucidation, the ^1^H-NMR spectrum did not show any protons related to NH or NH_2_ groups and displayed a singlet signal at *δ* 7.19 ppm for the CH group of pyridine ring. The ^13^C-NMR spectrum displayed a signal at *δ* 161.50 ppm for the carbonyl group. While the interaction of compound **11** with benzoyl chloride afforded a novel oxazinone derivative **13**. This cyclization was supported through the FT-IR spectrum that revealed an absorption peak at *ν* 1686 cm^−1^ related to the formation of the C = O group. Again, to confirm this elucidation, the ^1^H-NMR spectrum showed a singlet signal at *δ* 7.90 ppm related to the proton of pyridine ring. Furthermore, its ^13^C-NMR spectrum showed a signal at *δ* 167.89 ppm for the carbonyl group.

The enaminonitrile derivative **11** reacted with triethylorthoformate under reflux to yield the product **14**. The FT-IR spectrum of this derivative **14** showed lack absorption peaks for (NH_2_) group and absorption peak at *ν* 2217 cm^−1^ related to C≡N group. Moreover, the ^1^H-NMR spectrum displayed quartet and triplet signals at *δ* 4.42, and 1.39 ppm for CH_2_, and CH_3_ groups, respectively, beside a singlet signal at *δ* 7.77 ppm related to N＝CH group. The mass spectrum showed the molecular ion M^**∙ +**^ peak at *m/z* = 362 (33.10%).

### Biological activity

#### Antimicrobial evaluation

All synthesized compounds were screened for antimicrobial activity against a panel of representative microbial strains: two Gram-positive (*S. aureus*, *B. subtilis*), two Gram-negative (*E. coli*, *P. aeruginosa*), and one fungal strain (*C. albicans*). Minimum inhibitory concentrations (MICs) were determined using the broth microdilution method (Table 1) following CLSI guidelines.

**Table 1:** Evaluation of antimicrobial activity and MIC values of tested compounds.

Compound	Microbial strains							
	Gram negative			Gram positive				Fungi
	*E. coli*		*P. aeruginosa*		*S. aureus*	*B. subtilis*		*C. albicans*
1	50	50			25		25	50
2*	6.25	12.5			3.12		6.25	12.5
3*	6.25	6.25			3.12		6.25	6.25
4*	6.25	6.25			3.12		6.25	6.25
5*	6.25	12.5			3.12		6.25	12.5
6	12.5	25			12.5		12.5	25
7	50	50			25		25	50
8	25	50			25		25	50
9	25	50			12.5		25	25
10*	12.5	25			6.25		12.5	12.5
11	32	64			16		16	64
12	8	32			4		64	128
13*	4	16			2		32	64
14	16	64			8		128	128
Kn	–	–			–		–	2
OFX	2	4			1		1	–

MIC concentration in (µg/ml). Positive and negative control was (DMSO, microbial growth respectively). Ketoconazole (Kn) was used as a reference antifungal drug. Ofloxacin (OFX) was used as reference antibacterial drug. (

*) the most promising compounds.

Compounds **2**, **3**,** 4**,** 5**, **10**, and **13** exhibited the most potent and broad-spectrum activities, with MIC values ranging from 2 to 12.5 µg. mL^−1^ against Gram-positive and Gram-negative bacteria. Notably, compounds **3**,** 4**, and** 5** showed the lowest MICs (3.12 µg. mL^−1^ against *S. aureus*), suggesting their superior cell-wall penetration and enzyme inhibition potential. Compounds **10** and **13** also demonstrated remarkable activity against *E. coli* and *P. aeruginosa* (MIC = 12.5–16 µg. mL^−1^), indicating that halogen or nitro substitution enhances Gram-negative coverage. Thus, the present compounds possess antimicrobial potency comparable to or better than many structurally related N-heterocyclic derivatives reported in the literature^48,49^ as pyrazole and pyrazoline derivatives have demonstrated promising antimicrobial activity, with MIC values ranging from 6.25 to 12.5 µg/mL against *E. coli*, 12.5–32 µg/mL against *P. aeruginosa*, 3.12–8 µg/mL against *S. aureus*, and 12.5–64 µg/mL against *Candida albicans*. Overall, these reported analogues exhibit moderate to potent broad-spectrum antimicrobial activity, providing a suitable benchmark for evaluating the antimicrobial efficacy of the synthesized compounds in the present study.

*Checkerboard assay*: The checkerboard analysis demonstrated an indifferent interaction between the tested compounds and NAC, with FICI values of approximately 1.0, indicating that NAC neither enhanced nor antagonized the antibacterial activity of the compounds.

**Fig. 2:**
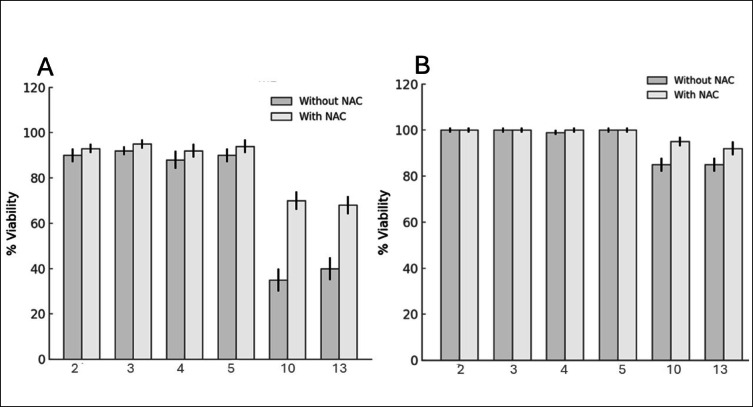
Effect of *N*-acetylcysteine (NAC) on the viability of HepG2 treated with tested compounds. Viability of cell line following 24 h exposure to compounds 2, 3, 4, 5, 10, and 13 (**A**) at concentration of 100 µg mL^−1^, While (**B**): at concentration of 5 µg mL^−1^, assessed in the absence (dark bars) and presence (light bars) of *N*-acetylcysteine (NAC). NAC supplementation markedly restored viability in cells treated with the cytotoxic compounds 10 and 13, whereas compounds 2–5 exhibited no significant cytotoxicity and showed viability values comparable to untreated controls. Data are presented as mean ± SD of three independent experiments.

*Effect of NAC on cytotoxicity*: The cytotoxic potential of selected compounds was evaluated against HepG2 cells using the MTT assay (Table 2, Fig. 2). At 100 µg. mL^−1^, most compounds (**2**,** 3**, **4**, and** 5**) showed high cell viability (> 88%), indicating low cytotoxicity. Compounds **10** and **13**, however, showed moderate cytotoxic effects with viabilities of 35% and 40%, respectively. In contrast to checkerboard, co-treatment with NAC significantly increased cell viability in the MTT assay, particularly for compounds **10** and **13**, suggesting that reactive oxygen species (ROS) contribute to their cytotoxic effects.

**Table 2:** Cytotoxicity of compounds against HepG2 Cells.

Compound	% Viability at 100 (µg. mL^−1^)	% Viability (+ NAC, 100 µg mL^−1^)	Folds of change	% Viability at 5 (µg. mL^−1^)	% Viability (+ NAC, 5 µg mL^−1^)	Folds of change
2	90 ± 3	93 ± 2	1.03	100 ± 1	100 ± 1	*NS
3	92 ± 2	95 ± 2	1.03	100 ± 1	100 ± 1	*NS
4	88 ± 4	92 ± 3	1.05	99 ± 1	100 ± 1	*NS
5	90 ± 3	94 ± 3	1.04	100 ± 1	100 ± 1	*NS
10	35 ± 5	70 ± 4	2	85 ± 3	95 ± 2	1.12
13	40 ± 5	68 ± 4	1.7	85 ± 3	92 ± 3	1.05

*NS means non-significant change. Fold-change = (% viability with NAC / % viability without NAC).

NAC co-treatment notably increased the viability of compounds **10** and **13**, while changes for the other compounds were non-significant (NS). Bars represent the mean ± SD of percentage cell viability between presence and absence of NAC.

Co-incubation with *N*-acetylcysteine (NAC) significantly reduced the cytotoxicity of compounds **10** and **13** (up to twofold increase in viability), suggesting that the antioxidant co-treatment mitigates oxidative stress-mediated damage. This observation aligns with the hypothesis that NAC can neutralize reactive oxygen intermediates generated by these compounds, thereby preserving cell viability without diminishing antimicrobial potency.

#### Anticancer potential

In addition to their antimicrobial evaluation, the cytotoxicity profiles of the synthesized compounds against the HepG2 cell line (a human hepatocellular carcinoma model) revealed valuable insights into their potential anticancer activity. Compounds **10** and **13** markedly reduced HepG2 cell viability to approximately 35–40% at 100 µg. mL^−1^, whereas compounds **2**,** 3**, **4**, and **5** maintained high cell viability (> 88%), confirming their relative biocompatibility. The pronounced cytotoxicity of compounds **10** and **13** suggests a possible antiproliferative effect on hepatic cancer cells, potentially mediated through reactive oxygen species (ROS) generation, as inferred from the observed protective effect of NAC co-treatment, which nearly doubled cell viability. This finding is consistent with the redox-active nature of nitro-substituted scaffolds; their strong electron-withdrawing character can promote the generation of reactive oxygen species, impair mitochondrial function, and induce apoptotic cell death. Accordingly, compounds **10** and **13** represent promising lead candidates for anticancer drug development. Future studies should focus on evaluating their selectivity toward cancer cells by determining their selectivity index, as well as investigating their ability to induce apoptosis and modulate ROS-dependent signaling pathways using a broader panel of both cancerous and normal cell lines.

#### Antibiofilm activity

**Fig. 3:**
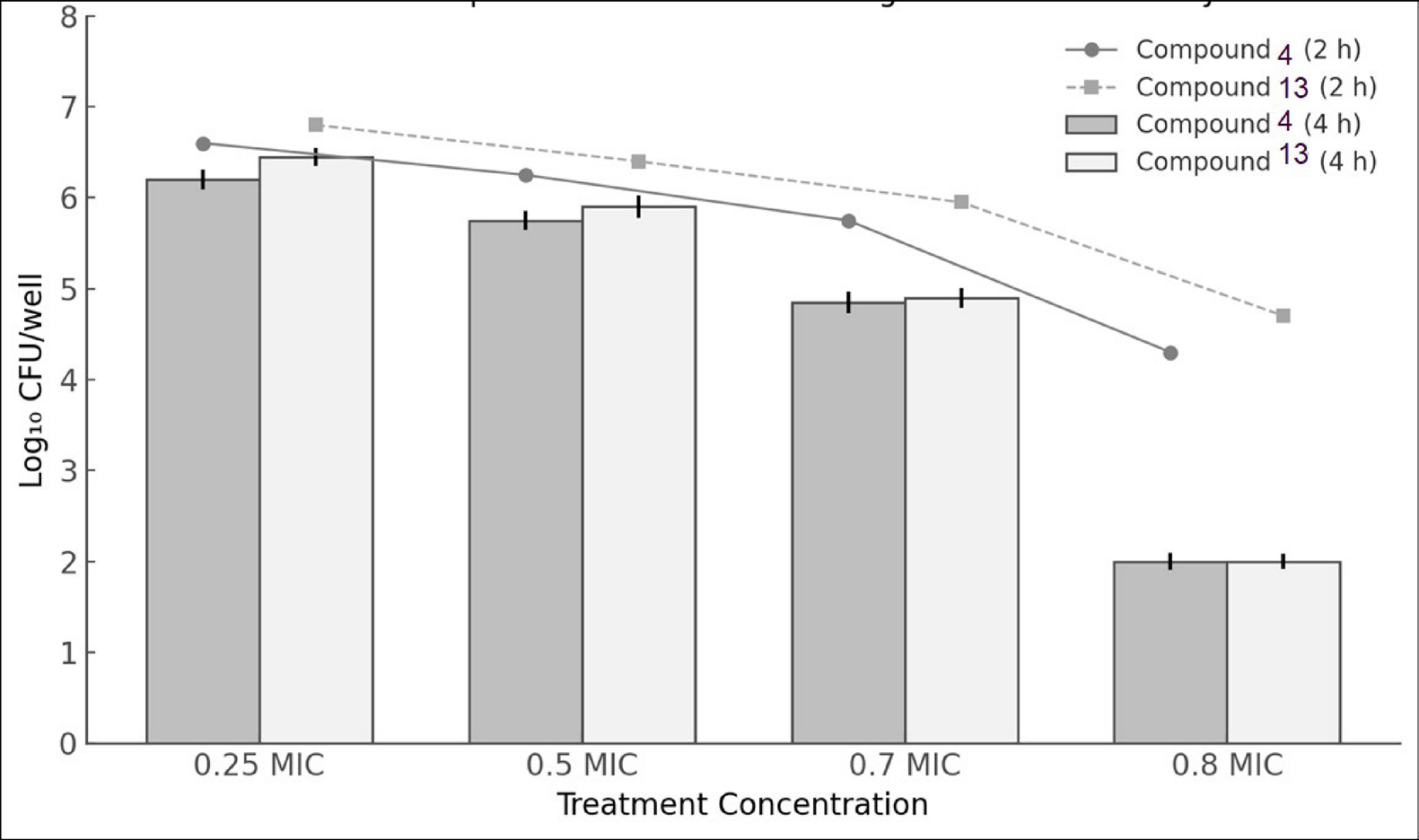
The effect of compounds **4** and **13** on the biofilm formation of *P. aeruginosa.*

Biofilm eradication assays revealed that both compound **4** and compound **13** effectively disrupted *P. aeruginosa* biofilms in a dose- and time-dependent manner (Table 3, Fig. 3). After 4 h of exposure at 0.8 MIC, both compounds achieved nearly complete eradication (> 5 log_10_ reduction; ≥ 99.999% reduction in viable cells). Even at sub-MIC concentrations (0.25–0.5 MIC), significant reductions (1–2 log_10_) were observed, indicating strong antibiofilm activity independent of complete bacterial killing. These results highlight the dual bactericidal and antibiofilm properties of these derivatives, making them promising candidates for treating persistent infections associated with *P. aeruginosa* biofilms.

**Table 3:** Antibiofilm activity of promising compounds.

Treatment		2 h	Log_10_ reduction	4 h	Log_10_ reduction	%Reduction after 4 h
PBS		7.45 ± 0.10	–	7.42 ± 0.08	–	–
0.25 MIC	Compound (4)	6.60 ± 0.11	0.85	6.20 ± 0.09	1.20	94.0%
	Compound (13)	6.80 ± 0.10	0.66	6.45 ± 0.08	0.98	90.6%
0.5 MIC	Compound (4)	6.25 ± 0.10	1.20	5.75 ± 0.08	1.65	97.8%
	Compound (13)	6.40 ± 0.12	1.06	5.90 ± 0.09	1.53	96.5%
0.7 MIC	Compound (4)	5.75 ± 0.12	1.70	4.85 ± 0.10	2.55	99.7%
	Compound (13)	5.95 ± 0.11	1.51	4.90 ± 0.10	2.53	99.7%
0.8 MIC	Compound (4)	4.30 ± 0.09	3.15	< 2.00	≥ 5.40	≥ 99.999%
	Compound (13)	4.70 ± 0.08	2.76	< 2.00	≥ 5.40	≥ 99.999%

Compound **4** significantly decreased viable biofilm cells in a dose- and time-dependent manner, achieving nearly complete eradication (> 5 log_10_ reduction) at 0.8 MIC after 4 h. Data are presented as mean ± SD of three independent biological experiments, each performed in triplicate. *Values expressed after 2 and 4 for log_10_ CFU/well (mean ± SD). **% Reduction is versus PBS. ***CPd: compound.

Bars represent 4-h biofilm viability, while the connected points show 2-h viability-clearly highlighting the dose- and time-dependent reduction in *P. aeruginosa* biofilms for both compounds. Data are presented as mean ± SD of three independent biological experiments, each performed in triplicate.

### Respiratory chain dehydrogenase (TTC) assay

The TTC assay results (Table 4) confirmed that compounds **4** and **13** severely impaired the metabolic activity of *P. aeruginosa* biofilms. The reduction of TTC to formazan—a measure of active dehydrogenase activity—decreased in a concentration- and time-dependent manner. After 4 h at ≥ 0.7 MIC, both compounds reduced metabolic activity to < 0.001% of control levels, indicating profound inhibition of bacterial respiratory chain enzymes.

**Table 4:** Effect of compounds 4 and 13 on Tetrazolium (TTC) Reduction in *Pseudomonas aeruginosa* Biofilms. Bacterial Viability (%) after treatment with compound 4 and compound 13 at different MIC fractions.

Treatment (MIC)	Compound 4		Compound 13	
	2 h	4 h	2 h	4 h
PBS (control)	100 ± 3	100 ± 3	100 ± 3	100 ± 3
0.25 MIC	14.1 ± 2.0%	6.31 ± 1.0%	21.9 ± 2.5%	10.5 ± 1.5%
0.5 MIC	6.31 ± 1.0%	2.24 ± 0.4%	8.71 ± 1.2%	2.95 ± 0.5%
0.7 MIC	2.00 ± 0.4%	0.28 ± 0.06%	3.09 ± 0.5%	0.30 ± 0.06%
0.8 MIC	0.071 ± 0.02%	< 0.001% (≤ 0.0004%)	0.174 ± 0.03%	< 0.001% (≤ 0.0004%)

Both compounds produced a pronounced, concentration and time-dependent reduction in tetrazolium reduction, indicating progressive inhibition of dehydrogenase-mediated metabolic activity. Compound **4** exhibited slightly greater early inhibition than compound **13**, as evidenced by the lower remaining metabolic activity after 2 h of treatment (e.g., 14.1% vs. 21.9% at 0.25 × MIC). Increasing the concentration and incubation time further reduced metabolic activity for both compounds, reaching near-background levels at ≥ 0.8 × MIC after 4 h. These findings are consistent with the CFU reduction results and indicate that both compounds markedly impair bacterial respiratory chain activity and cellular energy metabolism, with compound **4** exerting a slightly more rapid metabolic inhibitory effect. Data are presented as mean ± SD of three independent biological experiments, each performed in triplicate.

Compound **4** produced slightly greater early suppression of bacterial metabolic activity than compound **13**, as indicated by the lower remaining TTC reduction at 2 h (14.1% vs. 21.9% at 0.25 × MIC). This observation is consistent with the colony count data and suggests that compound** 4** more rapidly disrupts bacterial respiratory metabolism.

**Fig. 4:**
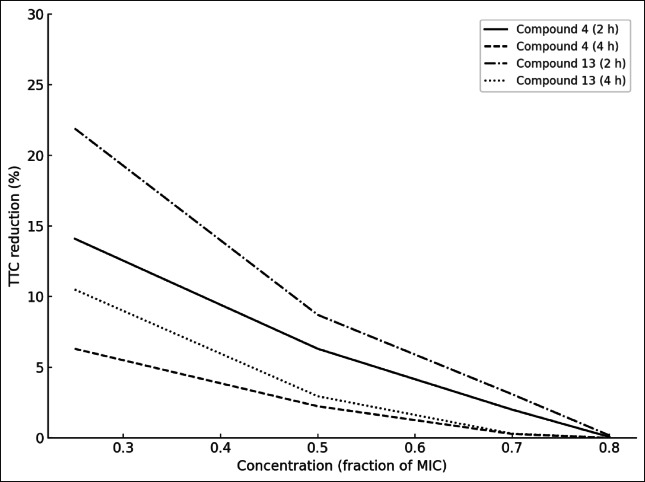
Effect of sub-MIC concentrations of compound 4 and compound 13 on bacterial metabolic activity assessed by TTC reduction assay at 2 h and 4 h incubation periods*.* Bacterial cells were exposed to compounds 4 or 13 at 0.5 × MIC, and metabolic activity was quantified by TTC reduction after 2 h (**A**) and 4 h (**B**) of incubation.

Bacterial cells were exposed to increasing fractions of the MIC (0.25, 0.5, 0.7, and 0.8 MIC) for each compound, and TTC reduction (%) was quantified relative to PBS-treated controls, as shown in Fig. 4. Both compounds exhibited a concentration- and time-dependent decrease in metabolic activity, with markedly enhanced reduction at higher MIC fractions. Compound **13** demonstrated a stronger inhibitory effect at early exposure (2 h), whereas Compound **4** showed more sustained inhibition at 4 h. At 0.8 MIC, both compounds reduced TTC activity to near-zero levels, indicating potent metabolic suppression. Values represent the mean ± standard deviation of three independent experiments.

### Molecular docking results and interpretation

Docking simulations revealed variable binding affinities among the compounds, ranging from –3.8 to –5.8 kcal/mol for *E. coli* gyrase and –3.7 to –5.4 kcal/mol for *S. aureus* gyrase. The co-crystallized ligands showed affinities of –5.8 and –4.6 kcal/mol, respectively, serving as reference points.

Several compounds demonstrated comparable or higher binding energies than the reference ligands. Notably, compounds **2**, **7**, **9**, and **11** exhibited strong interactions with key active site residues such as HIS83, ASP49, ARG76, and ARG84, forming multiple hydrogen bonds and salt bridges that may enhance binding stability. Distinct binding modes were observed between the two bacterial targets: *E. coli* gyrase complexes showed frequent hydrogen and salt bridge interactions with HIS83 and ARG76, while *S. aureus* gyrase primarily involved ARG84 and GLU58 interactions. Halogen bonding was detected in compound **3** with ASN46 of *E. coli* gyrase.

The docking analysis suggests that several of the synthesized compounds could act as promising inhibitors of bacterial DNA gyrase. The comparable or improved binding affinities relative to the co-crystallized ligands indicate strong potential for biological activity. The consistent involvement of residues HIS83, ARG76, and ARG84 across multiple compounds highlights their critical role in stabilizing ligand–enzyme complexes.

**Fig. 5:**
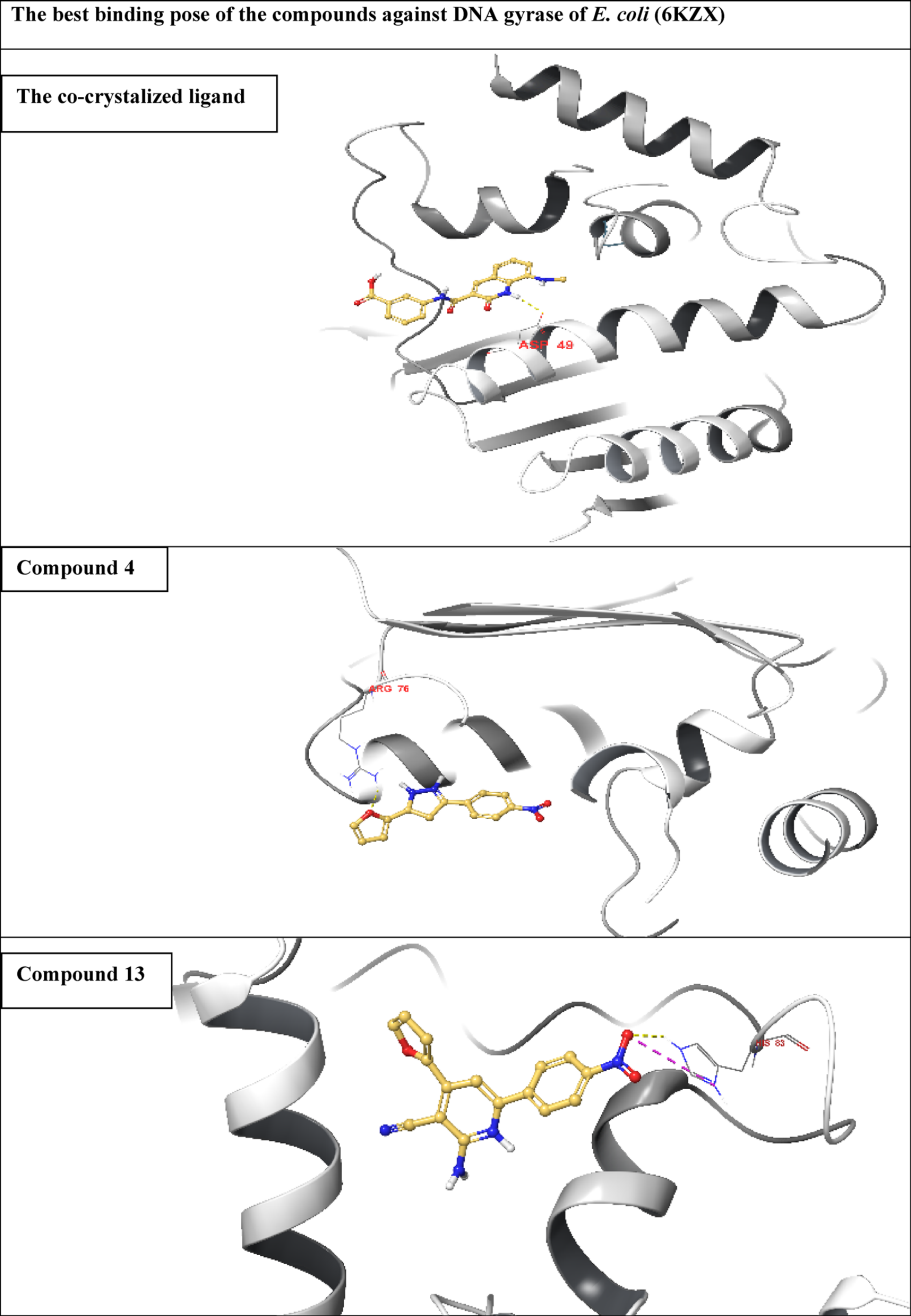
The best binding poses of the compounds against DNA gyrase of *E. coli* (6KZX).

**Fig. 6:**
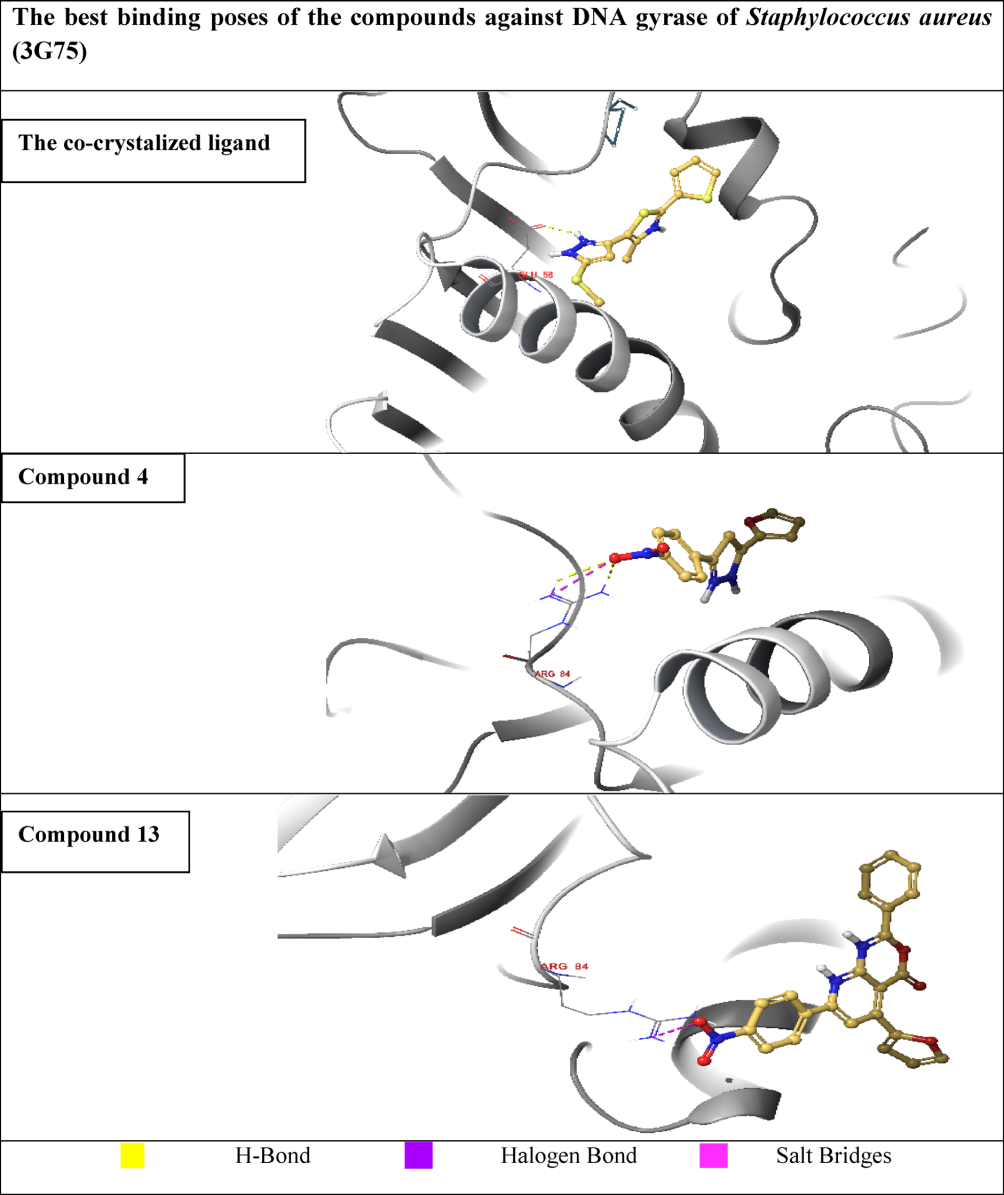
The best binding poses of the compounds against DNA gyrase of *Staphylococcus aureus* (3G75).

Compounds displaying dual hydrogen and salt bridge interactions, particularly **2**, **7**, **9**, and **11**, are predicted to possess enhanced inhibitory potential. Differences in binding profiles between *E. coli* and *S. aureus* targets underscore species-specific variations in the gyrase active site, which could guide future optimization for selectivity. Overall, the findings provide a molecular basis for further in *vitro* validation and structure–activity relationship (SAR) studies to confirm these compounds as lead candidates for antibacterial drug development (Figs. 5 and 6) (Table 5).

**Table 5:** Docking results of the compounds against DNA Gyrase of both *E. coli* and *Staphylococcus aureus.*

Compound	*E. coli* DNA Gyrase (6KZX)		*Staphylococcus aureus* DNA Gyrase (3G75)	
	Binding affinity (Kcal/mol)	Type of interaction and the interacted residues	Binding affinity (Kcal/mol)	Type of interaction and the interacted residues
Co-crystalized ligand	–5.8	H-Bond with ASP49	–4.6	H-Bond with GLU58
1	–5.4	H-Bond with ARG76	–4.9	H-Bond with ARG84
2	–5.7	H-Bond with ARG76	–5.4	2H-Bond and Salt Bridge with ARG84
3	–5.4	Salt Bridge with ARG76 Halogen bond with ASN 46	–4.6	2H-Bond and Salt Bridge with ARG84
4	–4.9	2 H-Bond with HIS83	–4.5	H-Bond with ARG84
5	–5.3	H-Bond with HIS83	–4.4	Salt Bridge and H-Bond with ARG84
6	–5.3	H-Bond with HIS83	–4.4	H-Bond with ARG84
7	–5.7	Salt Bridge with HIS83 H–Bond with ASP49	–4.8	Salt Bridge and H-Bond with ARG84
8	–3.8	Salt Bridge with ARG76	–4.1	Salt Bridge with ARG84
9	–5.6	Salt Bridge with HIS83 H-Bond with ASP49	–4.7	2H-Bond and Salt Bridge with ARG84
10	–4.9	H-Bond and Salt Bridge with HIS83	–4.6	Salt Bridge and H-Bond with ARG84
11	–5.8	H-Bond and Salt Bridge with HIS83	–4.3	Salt Bridge and H-Bond with ARG84
12	–5.2	2 H-Bond with GLU50	–4.8	2 H-Bond with ASP57 Salt Bridge with ARG84
13	–4.8	Salt Bridge with HIS83	–5	Salt Bridge with ARG84
14	–5	2 Salt Bridges with HIS55 and ARG76	–3.7	Salt Bridge and H-Bond with ARG84

### Transcriptional analysis of the DNA gyrase gene (gyrB)

Quantitative real-time PCR analysis demonstrated a marked downregulation of the gyrB gene in *E. coli* cells treated with the formula at 0.5 × MIC compared with untreated controls. Using rrsE as the internal reference and the ΔΔCt method, treated samples showed an average ΔΔCt of 2.2, corresponding to a ~ 4.6-fold decrease in gyrB expression (relative expression = 0.22 ± 0.05, *n* = 3, *p* = 0.004). This significant reduction indicates suppression of DNA gyrase activity, suggesting that the formula interferes with bacterial DNA replication and supercoiling. Furthermore, molecular docking results revealed strong ligand binding within the ATP-binding pocket of the GyrB subunit, reinforcing the molecular evidence that the compound complex impedes gyrase function. Collectively, these findings support a potential mechanism of antibacterial action involving inhibition of DNA gyrase at both transcriptional and structural levels. Although compounds **4** and **13** exhibited comparable antimicrobial activities, mechanistic validation by qRT-PCR was performed only for compound **4** because of its superior cytocompatibility. Therefore, the proposed DNA gyrase inhibition mechanism is currently supported only for compound **4**. Further studies should investigate whether compound **13** exerts a similar mechanism of action and extend transcriptional analyses to Gram-positive pathogens, particularly *Staphylococcus aureus*, to validate the broader applicability of the proposed mechanism (Table 6).

**Table 6:** The effect of compound 4 (0.5 × MIC) on gyrB Gene Expression in *E. coli.*

Sample	Ct (gyrB)	Ct (rrsE)	ΔCt (gyrB − rrsE)	ΔΔCt	Relative Expression (2^−^ΔΔCt)	Fold Change vs. Control	*p*-value
Control (untreated)	18.5 ± 0.3	12.0 ± 0.2	6.5	–	1.00	1.0 ×	–
Treated (0.5 × MIC)	20.8 ± 0.4	12.1 ± 0.1	8.7	2.2	0.22 ± 0.05	↓ 4.6-fold	0.004 **

Relative expression of the gyrB gene was quantified following treatment with compound 4 at 0.5 × the minimum inhibitory concentration (MIC) and compared with untreated control cells. Gene expression levels were normalized to the housekeeping gene rrsE (16S rRNA) and analyzed using the 2^ − ΔΔCt method.

## Experimental section

### Chemistry

**General:**

The Gallen Kamp melting point instrument was used to measure each melting point, and the results were uncorrected. A Pye-Unicam SP-3–300 infrared spectrophotometer was used to record the FTIR spectra, which were then represented in wavenumber (cm^–1^). Using TMS as an internal standard in deuterated dimethyl sulfoxide (DMSO-*d*_*6*_), ^1^H-NMR spectra were recorded at 300 and 400 MHz and ^13^C-NMR spectra at 100 MHz on a Varian Gemini spectrometer. Chemical changes were linked to the solvents and are expressed in *δ*. Every coupling constant (*J*) value is expressed in hertz. The units of chemical shifts (*δ*) are ppm. A CHN analyzer was used for elemental analysis, and every compound was within ± 0.4 of the theoretical values. Thin-layer chromatography (TLC) sheets coated with UV fluorescent silica gel Merck 60 F_254_ plates were used to monitor the reactions, and a UV lamp was used to observe the results.

#### Synthesis of 3-(furan-2-yl)-1-(4-nitrophenyl)prop-2-en-1-one (1)

With constant stirring, 10 ml of a 20% sodium hydroxide solution was added to a solution of* p*-nitroacetophenone (5 mmol, 0.82 g) in 15 ml of absolute ethanol. Furfural (5 mmol, 0.48 g) was then added to the reaction mixture. TLC was used to monitor the reaction, which was agitated overnight at 5–10 °C. The reaction liquid was put onto crushed ice and neutralized with HCl solution (1N) once the reaction was finished. The comparable pure product **1** was obtained as yellow crystals in a respectable yield of 88%, m.p. 140–142 °C, after the product was filtered and recrystallized from 1,4-dioxane. Anal. Calcd. for C_13_H_9_NO_4_ (243.05): C, 64.20; H, 3.73; N, 5.76. Found: C, 64.07; H, 3.46; N, 5.72. FTIR (KBr, *ν* cm^−1^): 1659 (C = O), 1587 (C = C). ^1^H-NMR (300 MHz, DMSO‑d_*6*_) *δ* (ppm): 8.34 (d, Ha, 2H *J* = 9.00 Hz), 8.25 (d, Hb, 2H, *J* = 9.00 Hz), 7.61 (d, Hd, 1H, *J* = 15.3 Hz), 7.49 (d, Hc, 1H, *J* = 15.6 Hz), 7.94–7.14 (m, 3H, Ar–H of furyl ring). MS (*m/z*, %): 243 (M^**∙ +**^; 17.52%).

#### Synthesis of 5-(furan-2-yl)-3-(4-nitrophenyl)-4,5-dihydro-*1H*-pyrazole (2)

Compound **1** (5 mmol, 1.22 g) and hydrazine hydrate (10 mmol, 0.49 g) were combined with ethanol (10 ml) and refluxed for six hours. After the reaction was completed, the reaction mixture was poured onto crushed ice and neutralized with 1N hydrochloric acid solution. A pure product **2** in the form of deep yellow crystals was obtained by collecting the solid product and recrystallizing it from ethanol, yield 82%, m.p. 122–124 °C. Anal. Calcd. for C_13_H_11_N_3_O_3_ (257.08): C, 60.70; H, 4.31; N, 16.33. Found: C, 60.58; H, 4.29; N, 16.07. FTIR (KBr, *ν* cm^−1^): 3335 (NH), 1593 (C = N). ^1^H-NMR (300 MHz, DMSO‑d_6_) *δ* (ppm): 8.26- 8.11 (m, 4H, Ar–H of 4-nitrophenyl ring), 7.83–6.36 (m, 3H, Ar–H of furyl ring), 7.18 (s, 1H, NH, exchangeable with D_2_O), 4.99 (t, 1H, CH-pyrazole), 3.40–3.07 (m, 2H, CH_2_). MS (*m/z*, %): 257 (M^**∙ +**^; 30.65%).

#### Synthesis of 2-chloro-1-(5-(furan-2-yl)-3-(4-nitrophenyl)-4,5-dihydro-*1H*-pyrazol-1-yl)ethan-1-one (3)

A catalytic amount of triethylamine (TEA) was present when compound **2** (10 mmol, 2.57 g) was dissolved in 20 ml of ethanol. The mixture was then refluxed for eight hours while ethyl chloroacetate (10 mmol, 1.1 mL) was added dropwise. After cooling, the reaction mixture was added to 50 ml of ice-cold water. By filtering, washing, and recrystallizing the resulting precipitate from ethanol in 48% yield, m.p. 231–233 °C. Compound **3** was produced as a yellow powder. Anal. Calcd. for C_15_H_12_ClN_3_O_4_ (333.7): C, 53.99; H, 3.62; N, 12.59. Found: C, 53.77; H, 3.34; N, 12.38. FT-IR (KBr, ν /cm^−1^): 3083 (CH_arom._), 2985, 2931 (CH_aliph_), 1693 (C = O). ^1^H-NMR (400 MHz, DMSO-*d*_*6*_) *δ* (ppm): 8.38–7.96 (m, 4H, 4-nitrophenyl ring), 7.59–6.57 (m, 3H, furyl ring), 5.63,5.60 (dd, 1H, (CH)pyrazole, *J* = 4.80 Hz, *J* = 12.00 Hz), 3.83, 3.79 (dd, 1H, (CH_2_)pyrazole, *J* = 4.80 Hz, *J* = 18.00 Hz), 3.71 (s, 2H, CH_2_Cl), 3.45, 3.41 (dd, 1H, (CH_2_)pyrazole, *J* = 4.80 Hz, *J* = 18.00 Hz). ^13^C-NMR (100 MHz, DMSO-*d*_6_) *δ* (ppm): 167.21 (C = O), 152.91, 148.36, 143.15, 137.43, 128.10, 126.41, 124.41, 111.11, 108.09, 55.53 (CH)pyrazole, 53.39 (CH_2_Cl), 38.59 (CH_2_)pyrazole.

#### Synthesis of 5-(furan-2-yl)-3-(4-nitrophenyl)-4,5-dihydro-*1H*-pyrazole-1-carbaldehyde (4)

Compound **1** (5 mmol, 1.22 g) and hydrazine hydrate (10 mmol, 0.49 g) were combined with formic acid (10 ml) and refluxed for nine hours under TLC monitoring. After cooling, the reaction mixture was transferred to crushed ice. Compound **4** was produced as weak yellow crystals after the precipitate was filtered, cleaned with water, dried, and recrystallized from 1,4-dioxane. Yield 75%, m.p. 164–166 °C. Anal. Calcd. for C_14_H_11_N_3_O_4_ (285.07): C, 58.95; H, 3.89; N, 14.73. Found: C, 58.78; H, 3.75; N, 14.57. FTIR (KBr, *ν* cm^−1^): 3117, 3067 (CH_aromatic_), 2921, 2851 (CH_aliphatic_), 1672 (C = O). ^1^H-NMR (400 MHz, DMSO‑d_6_) *δ* (ppm): 8.89 (s, 1H, CHO), 8.31- 8.01 (m, 4H, Ar–H of 4-nitrophenyl), 7.95- 6.91 (m, 3H, Ar–H of furyl), 5.68, 5.72 (dd, 1H, CH-pyrazole), 3.90–3.41 (m, 2H, CH_2_). ^13^C-NMR (100 MHz, DMSO-*d*_6_) *δ* (ppm): 160.68 (C = O), 155.17, 151.73, 148.64, 143.24, 137.01, 128.28, 124.47, 111.10, 108.38, 53.07 (CH)pyrazole, 38.80 (CH_2_)pyrazole. MS (*m/z*, %): 285 (M^**∙ +**^; 22.00%).

#### Synthesis of 5-(furan-2-yl)-3-(4-nitrophenyl)-*1H*-pyrazole-1-carboxylic acid (5)

A vigorous stirring solution of pyrazole derivative **4** (10 mmol, 3.01 g) in ethanol (20 mL) was combined with 50% aqueous NaOH solution (5 mL), and the combination was allowed to stir at room temperature for six hours. The course of the reaction was monitored using precoated TLC plates. The mixture was neutralized with 1N hydrochloric acid to produce the pure product as yellow crystals. The resulting precipitate was filtered and recrystallized from ethanol (m.p.: 238–240 °C, yield 61%). Anal. Calcd. for C_14_H_9_N_3_O_5_ (299.24): C, 56.19; H, 3.03; N, 14.04. Found: C, 56.11; H, 2.98; N, 13.94. FT-IR (KBr, ν /cm^−1^): br. 3441 (OH), 1680 (C = O). ^1^H-NMR (400 MHz, DMSO-*d*_*6*_) *δ* (ppm): 13.95 (s, 1H, OH, exchangeable with D_2_O), 8.35–8.13 (m, 4H, Ar–H, 4-nitrophenyl ring), 7.79 (s, 1H, pyrazole ring), 7.19- 6.62 (m, 3H, ArH of furyl ring). ^13^C-NMR (100 MHz, DMSO-*d*_6_) *δ* (ppm): 158.72 (C = O), 153.09, 146.97, 143.44, 141.11, 133.60, 126.52, 124.66, 112.34, 107.54, 100.79, 95.17.

#### Synthesis of 1-(5-(furan-2-yl)-3-(4-nitrophenyl)-4,5-dihydro-*1H*-pyrazol-1-yl)ethan-1-one (6)

**Method (A):**

Compound **1** (5 mmol, 1.22 g) and hydrazine hydrate (10 mmol, 0.49 g) were mixed with glacial acetic acid (10 ml) and refluxed for nine hours under TLC monitoring. After cooling, the reaction mixture was transferred onto crushed ice. Compound **6** was produced as pale-yellow crystals after the precipitate was filtered, cleaned with water, dried, and recrystallized from 1,4-dioxane. Produce 57%.

Method (B):

Compound **2** (10 mmol, 2.57 g) was refluxed for three hours using 5 ml of recently distilled acetic anhydride. TLC was used to advance the reaction. Following the completion of the reaction, the precipitate was filtered, dried, and recrystallized to produce compound **6** in a 75% yield at m.p. 164–166 °C. Anal. Calcd. for C_15_H_13_N_3_O_4_ (299.29): C, 60.20; H, 4.38; N, 14.04. Found: C, 59.82; H, 4.10; N, 13.65. FTIR (KBr, *ν* cm^−1^): 3117, 3067 (CH_aromatic_), 2921, 2851 (CH_aliphatic_), 1658 (C = O). ^1^H-NMR (300 MHz, DMSO‑d_6_) *δ* (ppm): 8.31- 8.01 (m, 4H, Ar–H of 4-nitrophenyl), 7.54- 6.35 (m, 3H, Ar–H of furyl), 5.72, 5.68 (dd, 1H, CH-pyrazole), 3.85–3.39 (m, 2H, CH_2_), 2.29 (s, 3H, CH_3_). MS (*m/z*, %): 299 (M^**∙ +**^; 34.12%).

#### Synthesis of 4-(furan-2-yl)-6-(4-nitrophenyl)pyrimidin-2-amine (7)

Compound **1** (5 mmol, 1.22 g) and guanidine hydrochloride (5 mmol, 0.30 g) were combined and refluxed for 12 h in an alcoholic potassium hydroxide solution (20% w/v, 10 ml). TLC tracked the development of reaction. Once the reaction was finished, it was cooled, put onto crushed ice, and acidified with diluted hydrochloric acid (1N). Product **7** was prepared by filtering out the precipitate, washing it with water, drying it, and recrystallizing it from ethanol^47^. Yield 77%; brown crystals; m.p. 220–222 °C. Anal. Calcd for: C_14_H_10_N_4_O_3_ (282.26): C, 59.57; H, 3.57; N, 19.85. Found: C, 59.29; H, 3.26; N, 19.52%. FTIR (KBr, *ν* cm^−1^): 3347, 3217 (NH_2_), 1654 (C = N), 1595 (C = C). MS (*m/z*, %): 282 (M^**∙ +**^; 16.14%).

#### Synthesis of 4-(furan-2-yl)-6-(4-nitrophenyl)-1,3-diphenyl-3,4-dihydropyrimidin-2(*1H*)-imine (8)

Compound **1** (5 mmol, 1.22 g) and 1,3-diphenylguanidine (5 mmol, 1.06 g) were combined and refluxed for 21 h in an alcoholic potassium hydroxide solution (20% w/v, 10 ml). TLC tracked the development of reaction. Once the reaction was finished, it was cooled, put onto crushed ice, and acidified with diluted hydrochloric acid (1N). A pure product** 8** in the form of deep brown crystals was obtained by filtering out the precipitate, washing it with water, drying it, and recrystallizing it from 1,4-dioxane. 49% yield; m.p. 160–162 °C.Anal. Calcd. for C_26_H_20_N_4_O_3_ (436.15): C, 71.55; H, 4.62; N, 12.84. Found: C, 71.42; H, 4.58; N, 12.72. FTIR (KBr, *ν* cm^−1^): br. 3200 (NH), 1649 (C = N), 1587 (C = C). ^1^H-NMR (300 MHz, DMSO‑*d*_*6*_) *δ* (ppm): 8.67 (s, 1H, NH, exchangeable by D_2_O), 8.18–6.97 (m, 17H, Ar–H), 6.56 (d, 1H, -CH of pyrimidine ring), 3.44 (d, 1H, = CH of pyrimidine). MS (*m/z*, %): 436 (M^**∙ +**^; 17.21%).

#### Synthesis of 6-(furan-2-yl)-4-(4-nitrophenyl)-5,6-dihydropyrimidin-2(*1H*)-one (9)

Compound **1** (5 mmol, 1.22 g) and urea (5 mmol, 0.30 g) were combined and refluxed for eighteen hours in an alcoholic potassium hydroxide solution (20% w/v, 10 mL). TLC tracked how the reaction was going. The reaction mixture was then cooled, poured onto crushed ice, and acidified with diluted hydrochloric acid (1N). Compound **9** was produced as deep brown crystals after the precipitate was filtered out, cleaned with water, dried, and recrystallized from ethanol. 65% yield; m.p.: 300–302 °C. Anal. Calcd for: C_14_H_11_N_3_O_4_ (285.26): C, 58.95; H, 3.89; N, 14.73. Found: C, 58.68; H, 3.52; N, 14.41%. FT-IR (KBr, *ν* cm^−1^): 3363 (NH), 3071 (CH_aromatic_), 1687 (C = O), 1600 (C = N). ^1^H-NMR (300 MHz, DMSO‑*d*_*6*_) *δ* (ppm): 8.36- 7.41 (m, 7H, Ar–H of 4-nitrophenyl and furyl rings), 6.57 (t, 1H, CH-pyrimidinone), 2.66 (d, 2H, CH_2_), 5.41 (s, 1H, NH, exchangeable with D_2_O). MS (*m/z*, %): 285 (M^**∙ +**^; 15.85%).

#### Synthesis of 1-allyl-6-(furan-2-yl)-4-(4-nitrophenyl)pyrimidine-2(*1H*)-thione (10)

Compound **1** (5 mmol, 1.22 g) and *N*-allylthiourea (5 mmol, 0.58 g) were combined in an alcoholic potassium hydroxide solution (20% w/v, 10 mL) and refluxed for 24 h. The development of reaction was tracked using TLC. When the reaction was finished, it was cooled, put onto crushed ice, and then acidified with diluted hydrochloric acid (1N). After filtering off the precipitate, it was cleaned with water, dried, and recrystallized from 1,4-dioxan to produce compound 10 as brown crystals. 57% yield; m.p.: 220–224 °C.Anal. Calcd for: C_17_H_13_N_3_O_3_S (339.07): C, 60.17; H, 3.86; N, 12.38. Found: C, 59.94; H, 3.78; N, 12.22. FT-IR (KBr, *ν* cm^−1^): 1684 (-CH = CH_2_), 1624 (C = N), 1215 (C = S). ^1^H-NMR (300 MHz, DMSO‑*d*_*6*_) *δ* (ppm): 8.38–8.10 (m, 4H, Ar–H of 4-nitrophenyl ring), 7.94–6.67 (m, 3H, Ar–H of furyl ring), 6.64 (s, 1H, CH-pyrimidine), 5.05–4.02 (br. m, 3H, -CH = CH_2_), 3.43 (d, 2H, -CH_2_-). MS (*m/z*, %): 339 (M^**∙ +**^; 13.72%).

#### Synthesis of 2-amino-4-(furan-2-yl)-6-(4-nitrophenyl)nicotinonitrile (11)

For 12 h, a combination of **1** (5 mmol, 1.22 g) and malononitrile (5 mmol, 0.33 g) in 15 ml of absolute ethanol with excess ammonium acetate was refluxed. The resultant precipitate was filtered out, cleaned with water, dried, and recrystallized from 1,4-dioxane to get a pure product **11** as a brown powder after the reaction liquid was cooled and placed onto crushed ice. 82% yield, m.p. 180–182 °C.Anal. Calcd. for C_16_H_10_N_4_O_3_ (306.08): C, 62.74; H, 3.29; N, 18.29. Found: C, 62.66; H, 3.17; N, 18.11. FTIR (KBr, *ν* cm^−1^): 3353–3241 (NH_2_), 2214 (C≡N), and 1662 (C = N). ^1^H-NMR (300 MHz, DMSO‑*d*_*6*_)* δ* (ppm): 8.40- 7.95 (m, 4H, Ar–H of 4-nitrophenyl), 7.91- 6.76 (m, 3H, Ar–H of furyl rings), 7.18 (s, 1H, pyridine ring), 6.90 (s, 2H, NH_2_, exchangeable with D_2_O). MS (*m/z*, %): 306 (M^**∙ +**^; 18.12%).

#### Synthesis of 5-(furan-2-yl)-7-(4-nitrophenyl)-*4H*-pyrido[2,3-*d*][1,3]oxazin-4-one (12)

Compound **11** (5 mmol, 1.53 g) and formic acid (10 mL) were refluxed for nine hours. The reaction mixture was added to water with crushed ice once it had cooled. Compound **12** was obtained as a white solid when the resultant solid was filtered off, cleaned with cold water, and recrystallized from ethanol. 75% yield, m.p. 202–205 °C. Anal. Calcd. for C_17_H_9_N_3_O_5_ (335.05): C, 60.90; H, 2.71; N, 12.53. Found: C, 60.76; H, 2.67; N, 12.31. FTIR (KBr, *ν* cm^−1^): 1714 (C = O) and 1618 (C = N). ^1^H-NMR (400 MHz, DMSO‑*d*_*6*_)* δ* (ppm): 8.39- 8.24 (m, 4H, Ar–H of 4-nitrophenyl), 7.92- 6.77 (m, 3H, Ar–H of furyl ring), 7.98 (s, 1H, CH-oxazinone ring), 7.19 (s, 1H, CH-pyridine ring). ^13^C-NMR (100 MHz, DMSO-*d*_6_) *δ* (ppm): 161.50 (C = O), 159.29, 154.82, 153.60, 149.21, 148.96, 148.35, 146.22, 144.13, 130.50, 124.19, 119.00, 116.06, 114.61, 105.86. MS (*m/z*, %): 335 (M^**∙ +**^; 47.19%).

#### Synthesis of 5-(furan-2-yl)-7-(4-nitrophenyl)-2-phenyl-*4H*-pyrido[2,3-d][1,3]oxazin-4-one (13)

For 24 h, compound **11** (5 mmol, 1.53 g) and benzoyl chloride (10 mL) were refluxed together. A rotating evaporator was used to eliminate the surplus benzoyl chloride. Deep brown crystals of product **13** were obtained by recrystallizing the solid residue from 1,4-dioxane following evaporation in yield (76%), m.p. 155–157 °C. Anal. Calcd. for C_23_H_13_N_3_O_5_ (411.4): C, 67.15; H, 3.19; N, 10.21. Found: C, 67.09; H, 3.03; N, 10.17. FTIR (KBr, *ν* cm^−1^): 1686 (C = O) and 1619 (C = N). ^1^H-NMR (400 MHz, DMSO‑*d*_*6*_)* δ* (ppm): 8.37- 7.94 (m, 4H, Ar–H of 4-nitrophenyl), 7.90 (s, 1H, pyridine ring), 7.61- 7.47 (m, 5H, Ar–H of phenyl ring), 7.17–6.77 (m, 3H, Ar–H of furyl ring). ^13^C-NMR (100 MHz, DMSO-*d*_6_) *δ* (ppm): 167.89 (C = O), 154.85, 149.02, 146.23, 144.17, 137.01, 133.28, 131.28, 130.53, 129.71, 129.01, 124.20, 114.64, 113.91, 113.46, 93.62.

#### Synthesis of ethyl-*N*-(3-cyano-4-(furan-2-yl)-6-(4-nitrophenyl)pyridin-2-yl)formimidate (14)

Compound **11** (5 mmol, 1.53 g) and 10 mL of triethyl orthoformate (TEOF) were refluxed for 24 h. TLC was used to track the progress of reaction, and the excess TEOF was vacuum-removed once it was finished. The residual solid was repeatedly cleaned with *n*-hexane and recrystallized from benzene to produce pale-yellow crystals of ethyl formimidate derivative **14**. 75% yield, m.p. 144–146 °C.Anal. Calcd. for C_19_H_14_N_4_O_4_ (362.35): C, 62.98; H, 3.89; N, 15.46. Found: C, 62.81; H, 3.91; N, 15.34. FTIR (KBr, *ν* cm^−1^): 2217 (C≡N), 1648 (C = N). ^1^H-NMR (300 MHz, DMSO‑*d*_*6*_) *δ* (ppm): 8.41–6.80 (m, 7H, Ar–H of 4-nitrophenyl and furyl rings), 8.03 (s, 1H, CH-pyridine ring), 7.77 (s, 1H, N＝CH), 4.42 (q, 2H, CH_2_, *J* = 6.9 Hz), 1.39 (t, 3H, CH_3_, *J* = 7.2 Hz). MS (*m/z*, %): 362 (M^**∙ +**^; 33.10%).

### Microbial strains and general growth conditions

In the present study, we used five microbial strains to screen the antimicrobial activity of synthesized compounds distributed as follows, two Gram *Bacillus subtilis (B. subtilis)* ATCC6051, *Staphylococcus aureus (S. aureus)* ATCC 9144, two Gram negative *Escherichia coli (E. coli)* O157:H7 ATCC 51,659 and *Pseudomonas aeruginosa* (*P. aeruginosa*) ATCC 27,853 and one fungal strains *Candida albicans* (*C. albicans*) ATCC 90,028.

Generally, Muller Hinton (MH) or Sabouraud dextrose agar and broth were used for bacterial and fungal growth respectively, at 37 °C for 24–30 h. Dulbecco’s Modified Eagle Medium (DMEM) enriched with 10% fetal bovine serum (FBS) and 0.1% antibiotic–antimycotic solution was used for culture of the HepG2 cell lines. Culture media and all reagents used were obtained from Sigma-Aldrich (USA), Oxoid (UK), or Fluka (Switzerland). All experiments were conducted in triplicate.

### Determination of minimum inhibitory concentration (MIC)

The antimicrobial potential of the synthesized compounds were evaluated by determination of minimum inhibitory concentrations (MICs) following the standard broth dilution method described by CLSI^50,51^. In summary, overnight cultures of reference microbial strains were diluted 1:1000 to obtain a final concentration of approximately 1.5 × 10^5^ CFU.mL^−1^. MIC values were assessed using a two-fold serial dilution across concentrations from 7.8 to 1000 µg. mL^−1^. The inoculated cultures were incubated at 37 °C with shaking at 150 rpm, and growth was monitored by measuring optical density at 600 nm. DMSO was used as control. All experiments were conducted in triplicate. Additional intermediate concentrations were tested (interdilution approach) to accurately determine the endpoint. The synergistic effect of the selected promising compounds in combination with *N*-acetylcysteine (NAC) was assessed using the checkerboard method to determine the fractional inhibitory concentration (FIC) index.

### Cytotoxicity testing

The Holding Company for Biological Products and Vaccines (VACSERA, Giza, Egypt) provided the HepG2 cell line used in this investigation. To investigate the cytotoxic effect of tested compounds, MTT (3-[4,5-dimethylthiazole-2-yl]-2,5-diphenyltetrazolium bromide) on HepG2 cell viability have been employed. In summary, 0.5 × 10^5^ cells/well in serum-free medium were plated in a flat bottom 96-well microplate and exposed to 20 µl of various doses 5–100 µg.mL^−1^ of the tested compounds over 48 h at 37º C in 5% CO_2_. Following 4 h of incubation, the media were withdrawn, 40 µl of MTT solution/well was applied, and the absorbance at 570 nm was measured photometrically using microplate reader (Biotek Elx-808)^51^.

### Antibiofilm activity

#### Determination of colony count

To determine the colony count of promising compounds **4** and **13**, biofilms were developed in 96-well plates by adding 100 μL of *Pseudomonas aeruginosa* suspension (10^6^ CFU/mL) to each well and allowing bacterial adhesion for 1 h. The suspension was then removed, and the wells were gently rinsed once with 100 μL of PBS. Afterwards, 200 μL of fresh growth medium was added, and the plates were incubated at 37 °C for 48 h^52^. Following incubation, the medium was discarded, and the wells were washed again with 100 μL of PBS. Treatments consisting of 100 μL PBS (control) or varying sub-MIC concentrations of the tested formulations were applied for 2 or 4 h. The biofilms were then disrupted by pipetting, serially diluted, and plated on LB agar^53^. Colony-forming units (CFU) were counted after incubation at 37 °C for 18 h. All assays were performed in triplicate.

### Potential antimicrobial activity

#### Assessment of respiratory chain dehydrogenases activity (TTC assay)

The respiratory chain dehydrogenase activity of *P. aeruginosa* biofilms was evaluated using a tetrazolium-based colorimetric assay (TTC or XTT), which reflects the metabolic reducing capacity of viable cells. After treatment with the tested compounds at different sub-MIC concentrations for the indicated times, cultures were exposed to the tetrazolium reagent at 37 °C for 3–4 h, allowing enzymatic reduction to the colored formazan product. The developed color intensity, corresponding to dehydrogenase activity, was measured spectrophotometrically using microplate reader (Biotek Elx-808) at 490 nm, after the incubation period. Control wells containing untreated cells and reagent blanks were included to correct for background absorbance. The metabolic activity for each treatment was expressed as a percentage relative to untreated control. All experiments were performed in triplicate, and data were presented as mean ± SD.

### In-Silico study

Molecular docking studies were carried out to evaluate the binding affinity and interaction modes of 14 synthetic compounds against the bacterial DNA gyrase enzyme, a key enzyme responsible for introducing negative supercoiling during DNA replication and transcription. AutoDock Vina, integrated within the PyRx 0.8 virtual screening platform, was employed for all docking experiments^54,55^. Two crystal structures were retrieved from the Protein Data Bank: *Escherichia coli* DNA gyrase subunit B (PDB ID: 6KZX) complexed with a quinoline derivative and *Staphylococcus aureus* DNA gyrase subunit B (PDB ID: 3G75) complexed with a thiazole inhibitor^56–58^. All water molecules were removed, and the co-crystallized ligands were retained to define the active site. Ligands were energy-minimized and converted into PDBQT format before docking. The grid box coordinates were centered at (x = 50.57, y = –3.38, z = 17.16) for 6KZX and (x = 27.02, y = 5.84, z = –8.37) for 3G75, with an exhaustiveness value of 20. The resulting docked complexes were analyzed and visualized using Schrödinger Maestro 14.6 (academic version) to study hydrogen bonding, salt bridges, and hydrophobic interactions within the active site. Docking protocol was validated by redocking the co-crystallized ligand, yielding an RMSD of 0.9 and 1.12 Å for *E. coli* and *S. aureus* respectively, between the predicted and experimental poses, confirming the reliability of the docking parameters. To validate the docking protocol, the co-crystallized ligand was redocked into the corresponding active site using the same docking parameters. The RMSD values between the crystallographic and redocked ligand poses were 0.90 Å for *E. coli* DNA gyrase (6KZX) and 1.12 Å for *S. aureus* DNA gyrase (3G75). Both values are below the generally accepted threshold of 2.0 Å, confirming that the docking protocol reliably reproduced the experimental binding mode and validating the docking methodology.

### Molecular targeting

Total RNA was extracted from *E. coli* cultures exposed to the formula at 0.5 × MIC and from untreated control groups using the RNeasy Mini Kit (Qiagen, Germany) following the manufacturer’s instructions. The purity and concentration of the isolated RNA were measured spectrophotometrically. Complementary DNA (cDNA) was synthesized from 1 µg of total RNA using a reverse transcription kit.

Quantitative real-time PCR (qRT-PCR) was then performed with gene-specific primers targeting the gyrB gene to evaluate the effect of the formula on its expression. The rrsE gene was used as a housekeeping reference for normalization. Amplification reactions were conducted in a real-time thermal cycler using SYBR Green Master Mix under optimized cycling conditions. Relative gene expression was calculated using the ΔΔCt method (Table 7).

**Table 7:** Sequence of primer used in qPCR.

Primer	Sequence	Amplicon
Forward	GAAAGCCGTTGGTGAAATGC	145 bp
Reverse	CGTTGTCGATACCAGCATCG	

## Structure activity relationship (SAR)

Based on the in vitro antimicrobial screening (Table 1), cytotoxicity profiles against HepG2 cells (Table 2), antibiofilm evaluation (Table 3), and molecular docking simulations (Tables 5, S43, S44), a comprehensive structure–activity relationship (SAR) was established for the synthesized derivatives. The starting chalcone scaffold, 3-(furan-2-yl)-1-(4-nitrophenyl)prop-2-en-1-one (**1**), which incorporates both an electron-withdrawing *p*-nitrophenyl ring and a lipophilic 2-furyl moiety, served as the foundational template. The investigated compounds were categorized into three distinct chemical classes based on the newly constructed heterocyclic rings: pyrazole derivatives (**2–6**), pyrimidine derivatives (**7–10**), and pyridine/oxazinone derivatives (**11–14**).

The following outlines the specific SAR discoveries into the impact of various substituents and heterocyclic frameworks:

- Impact of the Heterocyclic Frameworks: The newly formed central heterocycle’s characteristics were crucial in influencing the biological trajectory as a whole.
  Pyrazole ring: The MIC dropped from 25–50 µg/mL to 3.12–12.5 µg/mL as a result of the cyclization of the chalcone precursor **1** into the core pyrazole derivative **2**. This demonstrates that the nitrogen-containing pyrazole ring functions as an essential pharmacophore, perhaps improving binding selectivity and cellular absorption.
  Pyridone/Oxazinone fused system, fusing the pyridine core **11** into a bicyclic pyrido[2,3-d][1,3]oxazin-4-one architecture **12** and **13** profoundly amplified both the antimicrobial and anticancer profiles, outperforming the monocyclic pyrimidine core.
  Pyrimidine ring, conversely, monocyclic pyrimidines possessing a primary amino group (**7**), a diphenylimine core (**8**), or a carbonyl group (**9**) exhibited moderate-to-weak antimicrobial activity compared to the pyrazole family. This suggests that a saturated or fully aromatic pyrimidine core lacking highly reactive electrophilic handles possesses a lower capacity for cell-wall penetration.
- Influence of Substituents on Antimicrobial and Antibiofilm Activities: the electronic and steric properties of the substituents on the heterocyclic cores heavily dictated the antibacterial and antibiofilm efficacy:
  N-1 position of pyrazole and superiority of compound **4**: Introducing an N-acyl chloroacetyl (**3**) or a formyl group (**4**) at the N-1 position of the pyrazole core optimized the potency, yielding the highest efficacy against *S. aureus* (MIC = 3.12 µg/mL) along with broad Gram-negative coverage. While the presence of terminal reactive electrophilic groups facilitates targeted interactions within the bacterial enzymatic active sites (validated by compound **3** engaging in unique halogen bonding with the ASN46 residue of *E. coli* DNA gyrase), compound **4** emerged as a superior candidate due to its distinct mechanistic profile. The terminal reactive formyl (-CHO) group acts as a key electrophilic handle that precisely anchors inside the ATP-binding pocket of DNA gyrase. This structural advantage was translationally confirmed by qRT-PCR analysis, where compound **4** selectively induced a profound 4.6-fold downregulation of the gyrB gene expression in *E. coli* (*p* = 0.004). This dual transcriptional suppression and enzymatic blocking explain why compound **4** outperformed other pyrazole derivatives (such as the less reactive acetyl counterpart 6) in both raw antibacterial potency and rapid biofilm eradication.
  Steric Hindrance versus reactivity: Full aromatization with a carboxylic acid group (**5**) maintained excellent antibacterial potency (MIC = 3.12–12.5 µg/mL) while affording favorable biocompatibility (> 90% cell viability at 100 µg/mL). In contrast, the acetyl substitution (**6**) decreased activity against Gram-negative strains and fungi (MIC = 25 µg/mL), indicating that less reactive alkyl chains at the *N*-1 position create detrimental steric hindrance.
  Thione and Allyl Cooperativity: Within the pyrimidine class, the presence of a thione (C = S) group combined with an *N*-allyl substitution (**10**) significantly boosted antimicrobial behavior, displaying remarkable potency against *S. aureus* (MIC = 6.25 µg/mL) and *E. coli* (MIC = 12.5 µg/mL).
  Biofilm Eradication and mechanistic superiority of Oxazinone **13**: The introduction of a bulky, hydrophobic phenyl ring at the C-2 position of the fused oxazinone ring (**13**) generated an exceptional multi-targeting candidate with elite antibacterial activity (*S. aureus* MIC = 2 µg/mL; *E. coli* MIC = 4 µg/mL), significantly outperforming its precursor **11** and the monocyclic pyrimidines. In silico, compound **13** exhibited superior binding affinity against *S. aureus* DNA gyrase (-5.0 kcal/mol), outperforming the co-crystallized ligand (-4.6 kcal/mol) through a stable salt bridge with ARG84. Structurally, this extended hydrophobic framework facilitates exceptional passive diffusion across complex bacterial cell walls. Mechanistically, this high cellular uptake enables compound **13** to severely collapse the bacterial respiratory chain (reducing dehydrogenase-linked metabolic activity to < 0.001% in TTC assays), which seamlessly correlates with its elite capability (along with pyrazole **4**) to induce a ≥ 5 log_10_ reduction (≥ 99.999%) in *P. aeruginosa* viable biofilm cells after 4 h at 0.8 × MIC.
- Influence of Substituents on Anticancer and Cytotoxic Activities: The structural requirements for triggering cytotoxicity against HepG2 cancer cells were closely tied to lipophilicity and redox-activation:
  Oxidative Stress Trigger: the thione-bearing pyrimidine **10** exhibited strong cytotoxic potential against HepG2 cells (viability reduced to 35% at 100 µg/mL). The co-incubation with *N*-acetylcysteine (NAC) nearly doubled cell viability, confirming that the redox-active combination of the nitro-scaffold and the thione moiety collectively triggers oxidative stress via reactive oxygen species (ROS) generation.
  Hydrophobic Extension: similarly, the extended hydrophobic framework of the phenyl-substituted pyrido-oxazinone **13** augmented its cytotoxicity against HepG2 cells (40% cell viability), likely by facilitating passive diffusion across the cancer cell membranes and anchoring deeper into the lipophilic pockets of its cellular targets.

## Conclusion

In conclusion, a strategic chalcone scaffold was used to successfully synthesize a novel range of functionalized pyrazole, pyrimidine, and pyridine/oxazinone derivatives. Strong multi-targeting compounds with exceptional dual antibacterial and anticancer capabilities were produced by the synergistic interaction between the electron-withdrawing nitrophenyl moieties and the lipophilic furan. The most promising therapeutic leads were found to be the fused pyrido-oxazinone framework (**13**) and the *N*-formyl pyrazole core (**4**). By interfering with bacterial respiratory chain dehydrogenase enzymes, these drugs completely eliminated persistent *P. aeruginosa* biofilms and showed remarkable antibacterial effectiveness against *S. aureus*. Additionally, compounds **10** and **13** demonstrated strong and specific cytotoxicity against HepG2 cancer cells through an apoptotic mechanism mediated by reactive oxygen species (ROS), whilst the remaining pyrazole core derivatives showed good biocompatibility profiles. In silico molecular docking studies within the ATP-binding pocket of DNA gyrase was closely linked with qRT-PCR transcriptional downregulation of the bacterial gyrB gene, which mechanistically verified the targeted antibacterial effect. All of these results point to these specific heterocycles being extremely powerful, dual-acting templates. Determining the exact selectivity index (SI) in non-cancerous cell lines, expanding the screening panel against clinical multidrug-resistant isolates, and performing in vivo preclinical validation for advanced drug development will be the main goals of future perspectives.

## Supplementary Information

Below is the link to the electronic supplementary material.

Supplementary Material 1

## Data Availability

All data generated or analyzed during this study are included in this published article and its supplementary information files.
