# Supplementary material for: Synthesis and molecular docking of novel pyrazole, pyrimidine, and pyridine derivatives as potent antimicrobial, antibiofilm, and anticancer agents
Source: Sci Rep. 2026 Aug 8;16:24543. doi: 10.1038/s41598-026-65144-w (PMC13452809; doi:10.1038/s41598-026-65144-w)
Supplement: Supplementary file 1 [file 41598_2026_65144_MOESM1_ESM.docx]

**Synthesis and Molecular Docking of Novel Pyrazole, Pyrimidine, and Pyridine Derivatives as Potent Antimicrobial, Antibiofilm, and Anticancer Agents**

**A.M.A. Hassan^1, *^, Essam E. S.^2^, Selwan Hamed ^3^, M. H. Helal^4^, A.K. El-Ziaty^1^ and Rania S. Ali^2^**

^1^Department of Chemistry, Faculty of Science, Ain Shams University, Abbassia 11566, Cairo, Egypt.

^2^Department of Basic Science, Faculty of Technology and Education, Capital University, Cairo, Egypt.

^3^Department of Microbiology and Immunology, Faculty of Pharmacy, Capital University, Ain Helwan, Helwan, 11795, Egypt.

^4^Department of Chemistry, Faculty of Science, Capital University, Ain Helwan, Cairo, Egypt.

**^*^** Corresponding author:

E-mail: [*ayman.mohammed@sci.asu.edu.eg*](mailto:ayman.mohammed@sci.asu.edu.eg)

**Table of Contents**

| **Title** | **Page** |
| --- | --- |
| **FT-IR, ^1^H NMR, ^13^C NMR, and mass spectral data of targets (1-14)** | |
| **Figure S1.** FTIR spectrum of compound **1** | **S4** |
| **Figure S2.** ^1^H-NMR (300 MHz, DMSO-*d_6_*) spectrum of compound **1** | **S5** |
| **Figure S3.** Mass spectrum of compound **1** | **S6** |
| **Figure S4.** FTIR spectrum of compound **2** | **S7** |
| **Figure S5.** ^1^H-NMR (300 MHz, DMSO-*d_6_*) spectrum of compound **2** | **S8** |
| **Figure S6.** Mass spectrum of compound **2** | **S9** |
| **Figure S7.** FTIR spectrum of compound **3** | **S10** |
| **Figure S8.** ^1^H-NMR (400 MHz, DMSO-*d_6_*) spectrum of compound **3** | **S11** |
| **Figure S9.** ^13^C-NMR (100 MHz, DMSO-*d_6_*) spectrum of compound **3** | **S12** |
| **Figure S10.** FTIR spectrum of compound **4** | **S13** |
| **Figure S11.** ^1^H-NMR (400 MHz, DMSO-*d_6_*) spectrum of compound **4** | **S14** |
| **Figure S12.** ^13^C-NMR (100 MHz, DMSO-*d_6_*) spectrum of compound **4** | **S15** |
| **Figure S13.** Mass spectrum of compound **4** | **S16** |
| **Figure S14.** FTIR spectrum of compound **5** | **S17** |
| **Figure S15.** ^1^H-NMR (400 MHz, DMSO-*d_6_*) spectrum of compound **5** | **S18** |
| **Figure S16.** ^13^C-NMR (100 MHz, DMSO-*d_6_*) spectrum of compound **5** | **S19** |
| **Figure S17.** FTIR spectrum of compound **6** | **S20** |
| **Figure S18.** ^1^H-NMR (300 MHz, DMSO-*d_6_*) spectrum of compound **6** | **S21** |
| **Figure S19.** Mass spectrum of compound **6** | **S22** |
| **Figure S20.** FTIR spectrum of compound **7** | **S23** |
| **Figure S21.** Mass spectrum of compound **7** | **S24** |
| **Figure S22.** FTIR spectrum of compound **8** | **S25** |
| **Figure S23.** ^1^H-NMR (300 MHz, DMSO-*d_6_*) spectrum of compound **8** | **S26** |
| **Figure S24.** Mass spectrum of compound **8** | **S27** |
| **Figure S25.** FTIR spectrum of compound **9** | **S28** |
| **Figure S26.** ^1^H-NMR (300 MHz, DMSO-*d_6_*) spectrum of compound **9** | **S29** |
| **Figure S27.** Mass spectrum of compound **9** | **S30** |
| **Figure S28.** FTIR spectrum of compound **10** | **S31** |
| **Figure S29.** ^1^H-NMR (300 MHz, DMSO-*d_6_*) spectrum of compound **10** | **S32** |
| **Figure S30.** Mass spectrum of compound **10** | **S33** |
| **Figure S31.** FTIR spectrum of compound **11** | **S34** |
| **Figure S32.** ^1^H-NMR (300 MHz, DMSO-*d_6_*) spectrum of compound **11** | **S35** |
| **Figure S33.** Mass spectrum of compound **11** | **S36** |
| **Figure S34.** FTIR spectrum of compound **12** | **S37** |
| **Figure S35.** ^1^H-NMR (400 MHz, DMSO-*d_6_*) spectrum of compound **12** | **S38** |
| **Figure S36.** ^13^C-NMR (100 MHz, DMSO-*d_6_*) spectrum of compound **12** | **S39** |
| **Figure S37.** Mass spectrum of compound **12** | **S40** |
| **Figure S38.** FTIR spectrum of compound **13** | **S41** |
| **Figure S39.** ^1^H-NMR (400 MHz, DMSO-*d_6_*) spectrum of compound **13** | **S42** |
| **Figure S40.** ^13^C-NMR (100 MHz, DMSO-*d_6_*) spectrum of compound **13** | **S43** |
| **Figure S41.** FTIR spectrum of compound **14** | **S44** |
| **Figure S42.** ^1^H-NMR (300 MHz, DMSO-*d_6_*) spectrum of compound **14** | **S45** |
| **Figure S43. The best binding poses of the compounds against DNA gyrase of *E. coli* (6KZX)** | **S47** |
| **Figure S44. The best binding poses of the compounds against DNA gyrase of *Staphylococcus aureus* (3G75)** | **S52** |

**FT-IR, ^1^H-NMR, ^13^C-NMR, Mass spectral data of targets (1-12)**

**Figure S1.** FTIR spectrum of compound **1**

**Figure S2.** ^1^H-NMR (300 MHz, DMSO-*d_6_*) spectrum of compound **1**

**Figure S3.** Mass spectrum of compound **1**

**Figure S4.** FTIR spectrum of compound **2**

**Figure S5.** ^1^H-NMR (300 MHz, DMSO-*d_6_*) spectrum of compound **2**

**Figure S6.** Mass spectrum of compound **2**

**Figure S7.** FTIR spectrum of compound **3**

**Figure S8.** ^1^H-NMR (400 MHz, DMSO-*d_6_*) spectrum of compound **3**

**Figure S9.** ^13^C-NMR (100 MHz, DMSO-*d_6_*) spectrum of compound **3**

**Figure S10.** FTIR spectrum of compound **4**

**Figure S11.** ^1^H-NMR (400 MHz, DMSO-*d_6_*) spectrum of compound **4**

**Figure S12.** ^13^C-NMR (100 MHz, DMSO-*d_6_*) spectrum of compound **4**

**Figure S13.** Mass spectrum of compound **4**

**Figure S14.** FTIR spectrum of compound **5**

**Figure S15.** ^1^H-NMR (400 MHz, DMSO-*d_6_*) spectrum of compound **5**

**Figure S16.** ^13^C-NMR (100 MHz, DMSO-*d_6_*) spectrum of compound **5**

**Figure S17.** FTIR spectrum of compound **6**

**Figure S18.** ^1^H-NMR (300 MHz, DMSO-*d_6_*) spectrum of compound **6**

**Figure S19.** Mass spectrum of compound **6**

**Figure S20.** FTIR spectrum of compound **7**

**Figure S21.** Mass spectrum of compound **7**

**Figure S22.** FTIR spectrum of compound **8**

**Figure S23.** ^1^H-NMR (300 MHz, DMSO-*d_6_*) spectrum of compound **8**

**Figure S24.** Mass spectrum of compound **8**

**Figure S25.** FTIR spectrum of compound **9**

**Figure S26.** ^1^H-NMR (300 MHz, DMSO-*d_6_*) spectrum of compound **9**

**Figure S27.** Mass spectrum of compound **9**

**Figure S28.** FTIR spectrum of compound **10**

**Figure S29.** ^1^H-NMR (300 MHz, DMSO-*d_6_*) spectrum of compound **10**

**Figure S30.** Mass spectrum of compound **10**

**Figure S31.** FTIR spectrum of compound **11**

**Figure S32.** ^1^H-NMR (300 MHz, DMSO-*d_6_*) spectrum of compound **11**

**Figure S33.** Mass spectrum of compound **11**

**Figure S34.** FTIR spectrum of compound **12**

**Figure S35.** ^1^H-NMR (400 MHz, DMSO-*d_6_*) spectrum of compound **12**

**Figure S36.** ^13^C-NMR (100 MHz, DMSO-*d_6_*) spectrum of compound **12**

**Figure S37.** Mass spectrum of compound **12**

**Figure S38.** FTIR spectrum of compound **13**

**Figure S39.** ^1^H-NMR (400 MHz, DMSO-*d_6_*) spectrum of compound **13**

**Figure S40.** ^13^C-NMR (100 MHz, DMSO-*d_6_*) spectrum of compound **13**

**Figure S41.** FTIR spectrum of compound **14**

**Figure S42.** ^1^H-NMR (300 MHz, DMSO-*d_6_*) spectrum of compound **14**

**Figure S43.** Mass spectrum of compound **14**

**Figure S43. The best binding poses of the compounds against DNA gyrase of *E. coli* (6KZX)**

| **The best binding pose of the compounds against DNA gyrase of *E. coli* (6KZX)** | |
| --- | --- |
| **The co-crystalized ligand** | **Compound 1** |
|  |  |
| **Compound 2** | **Compound 3** |
|  |  |
| **Compound 4** | **Compound 5** |
|  |  |
| **Compound 6** | **Compound 7** |
|  |  |
| **Compound 8** | **Compound 9** |
|  |  |
| **Compound 10** | **Compound 11** |
|  |  |
| **Compound 12** | **Compound 13** |
|  |  |
| **Compound 14** |  |
|  |  |
|  \| H-Bond \|  \| Halogen Bond \|  \| Salt Bridges | |

**Figure S44.** The best binding poses of the compounds against DNA gyrase of *Staphylococcus aureus* (3G75)

| **The best binding poses of the compounds against DNA gyrase of Staphylococcus aureus (3G75)** | |
| --- | --- |
| **The co-crystalized ligand** | **Compound 1** |
|  |  |
| **Compound 2** | **Compound 3** |
|  |  |
| **Compound 4** | **Compound 5** |
|  |  |
| **Compound 6** | **Compound 7** |
|  |  |
| **Compound 8** | **Compound 9** |
|  |  |
| **Compound 10** | **Compound 11** |
|  |  |
| **Compound 12** | **Compound 13** |
|  |  |
| **Compound 14** |  |
|  |  |
|  \| H-Bond \|  \| Halogen Bond \|  \| Salt Bridges | |
